# Temperature synchronizes temporal variation in laying dates across European hole‐nesting passerines

**DOI:** 10.1002/ecy.3908

**Published:** 2022-12-21

**Authors:** Stefan J. G. Vriend, Vidar Grøtan, Marlène Gamelon, Frank Adriaensen, Markus P. Ahola, Elena Álvarez, Liam D. Bailey, Emilio Barba, Jean‐Charles Bouvier, Malcolm D. Burgess, Andrey Bushuev, Carlos Camacho, David Canal, Anne Charmantier, Ella F. Cole, Camillo Cusimano, Blandine F. Doligez, Szymon M. Drobniak, Anna Dubiec, Marcel Eens, Tapio Eeva, Kjell Einar Erikstad, Peter N. Ferns, Anne E. Goodenough, Ian R. Hartley, Shelley A. Hinsley, Elena Ivankina, Rimvydas Juškaitis, Bart Kempenaers, Anvar B. Kerimov, John Atle Kålås, Claire Lavigne, Agu Leivits, Mark C. Mainwaring, Jesús Martínez‐Padilla, Erik Matthysen, Kees van Oers, Markku Orell, Rianne Pinxten, Tone Kristin Reiertsen, Seppo Rytkönen, Juan Carlos Senar, Ben C. Sheldon, Alberto Sorace, János Török, Emma Vatka, Marcel E. Visser, Bernt‐Erik Sæther

**Affiliations:** ^1^ Centre for Biodiversity Dynamics, Department of Biology Norwegian University of Science and Technology Trondheim Norway; ^2^ Department of Animal Ecology Netherlands Institute of Ecology (NIOO‐KNAW) Wageningen The Netherlands; ^3^ Laboratoire de Biométrie et Biologie Evolutive UMR 5558, CNRS Université Claude Bernard Lyon 1 Villeurbanne France; ^4^ Evolutionary Ecology Group, Department of Biology University of Antwerp Antwerp Belgium; ^5^ Environmental Research and Monitoring Swedish Museum of Natural History Stockholm Sweden; ^6^ Ecology of Terrestrial Vertebrates, ‘Cavanilles’ Institute of Biodiversity and Evolutionary Biology University of Valencia Valencia Spain; ^7^ Department of Evolutionary Genetics Leibniz Institute for Zoo and Wildlife Research (IZW) in the Forschungsverbund Berlin e.V Berlin Germany; ^8^ INRAE, Plantes et Systèmes de culture Horticoles Avignon France; ^9^ RSPB Centre for Conservation Science Sandy UK; ^10^ Centre for Research in Animal Behaviour University of Exeter Exeter UK; ^11^ Department of Vertebrate Zoology Moscow State University Moscow Russia; ^12^ Department of Biological Conservation and Ecosystem Restoration Pyrenean Institute of Ecology (IPE‐CSIC) Jaca Spain; ^13^ Institute of Ecology and Botany, Centre for Ecological Research Vácrátót Hungary; ^14^ CEFE, Univ Montpellier, CNRS, EPHE, IRD Montpellier France; ^15^ Department of Zoology, Edward Grey Institute University of Oxford Oxford UK; ^16^ Stazione Ornitologica Aegithalos Monreale Italy; ^17^ Department of Ecology and Genetics/Animal Ecology Uppsala University Uppsala Sweden; ^18^ Institute of Environmental Sciences Jagiellonian University Krakow Poland; ^19^ Evolution & Ecology Research Centre, School of Biological, Environmental and Earth Sciences University of New South Wales Sydney New South Wales Australia; ^20^ Museum and Institute of Zoology, Polish Academy of Sciences Warsaw Poland; ^21^ Behavioural Ecology & Ecophysiology Group, Department of Biology University of Antwerp Wilrijk Belgium; ^22^ Department of Biology University of Turku Turku Finland; ^23^ Kevo Subarctic Research Institute University of Turku Turku Finland; ^24^ Norwegian Institute for Nature Research (NINA), FRAM High North Research Centre for Climate and the Environment Tromsø Norway; ^25^ Cardiff School of Biosciences Cardiff University Cardiff UK; ^26^ School of Natural and Social Sciences University of Gloucestershire Cheltenham UK; ^27^ Lancaster Environment Centre Lancaster University Lancaster UK; ^28^ Centre for Ecology and Hydrology Wallingford UK; ^29^ Zvenigorod Biological Station Moscow State University Moscow Russia; ^30^ Nature Research Centre Vilnius Lithuania; ^31^ Department of Behavioural Ecology and Evolutionary Genetics Max Planck Institute for Ornithology Seewiesen Germany; ^32^ Department of Terrestrial Ecology Norwegian Institute for Nature Research (NINA) Trondheim Norway; ^33^ Department of Nature Conservation Environmental Board Saarde Estonia; ^34^ Ecology and Genetics Research Unit University of Oulu Oulu Finland; ^35^ Research Group Didactica, Antwerp School of Education University of Antwerp Antwerp Belgium; ^36^ Evolutionary and Behavioural Ecology Research Unit Museu de Ciències Naturals de Barcelona Barcelona Spain; ^37^ Institute for Environmental Protection and Research Rome Italy; ^38^ Behavioural Ecology Group, Department of Systematic Zoology and Ecology Eötvös Loránd University (ELTE) Budapest Hungary; ^39^ Ecological Genetics Research Unit, Organismal and Evolutionary Biology Research Programme University of Helsinki Helsinki Finland

**Keywords:** birds, climate, clutch size, comparative analysis, fitness‐related traits, fledgling number, phenology, spatial synchrony, timing of breeding, weather

## Abstract

Identifying the environmental drivers of variation in fitness‐related traits is a central objective in ecology and evolutionary biology. Temporal fluctuations of these environmental drivers are often synchronized at large spatial scales. Yet, whether synchronous environmental conditions can generate spatial synchrony in fitness‐related trait values (i.e., correlated temporal trait fluctuations across populations) is poorly understood. Using data from long‐term monitored populations of blue tits (*Cyanistes caeruleus*, *n* = 31), great tits (*Parus major*, *n* = 35), and pied flycatchers (*Ficedula hypoleuca*, *n* = 20) across Europe, we assessed the influence of two local climatic variables (mean temperature and mean precipitation in February–May) on spatial synchrony in three fitness‐related traits: laying date, clutch size, and fledgling number. We found a high degree of spatial synchrony in laying date but a lower degree in clutch size and fledgling number for each species. Temperature strongly influenced spatial synchrony in laying date for resident blue tits and great tits but not for migratory pied flycatchers. This is a relevant finding in the context of environmental impacts on populations because spatial synchrony in fitness‐related trait values among populations may influence fluctuations in vital rates or population abundances. If environmentally induced spatial synchrony in fitness‐related traits increases the spatial synchrony in vital rates or population abundances, this will ultimately increase the risk of extinction for populations and species. Assessing how environmental conditions influence spatiotemporal variation in trait values improves our mechanistic understanding of environmental impacts on populations.

## INTRODUCTION

Understanding spatial and temporal variation in traits is a central objective in ecology and evolutionary biology (Berven & Gill, 1983; Jetz et al., 2008; Lack, 1947; Moreau, 1944; Ruuskanen et al., 2011). Particular attention has been directed at understanding variation in traits that directly link to fitness. Fitness‐related traits (sometimes more generally referred to as functional traits) can be defined as measurable traits that impact individual fitness (e.g., body mass, timing of breeding, offspring number; Violle et al., 2007). Key to improving our understanding of spatial and temporal variation in such traits is identifying the environmental variables that drive them and examining how variation in these environmental drivers relates to variation in fitness‐related traits.

Among the best studied fitness‐related traits are timing of breeding, clutch size, and fledgling number in birds. Arguably the most striking spatial pattern in the values of these traits are latitudinal gradients (Lack, 1947; Moreau, 1944). With increasing latitudes, breeding tends to start later and clutch size and fledgling number tend to increase (Bailey et al., 2022; Sanz, 1997). These traits also vary substantially across years. Annual variation in the timing of breeding, clutch size, and fledgling number has been linked to various environmental variables, including timing and availability of resources (Visser et al., 2006), breeding density (Dunn & Winkler, 2010), temperature (Sanz et al., 2003), precipitation (Öberg et al., 2015), and large‐scale weather indices like the North Atlantic Oscillation (NAO) index (Møller, 2002), as well as the interaction between environmental variables (Møller et al., 2020). Further, within seasons, clutch size and fledgling number may be affected by the timing of breeding; they generally decrease with later laying (Perrins, 1970), and this seasonal decline is stronger at higher latitudes (Winkler et al., 2014). However, the link between laying date and clutch size or fledgling number across years is unclear. Depending on the trait under study, the influence of environmental variables may differ between species, habitats, or geographic locations. For example, timing of breeding in birds and other taxa across the globe has advanced in response to increasing temperatures (Dunn & Winkler, 2010) but to varying degrees among species and geographic areas (Bailey et al., 2022; Visser et al., 2003). The responses of clutch size and fledgling number to increasing temperatures, however, are not so straightforward. Clutch size may increase with increasing temperatures (Both & Visser, 2005), decrease (Laaksonen et al., 2006), or display no temperature‐related fluctuations (Husby et al., 2010). Further, in some populations, laying date did not respond to increasing temperatures, whereas clutch size and fledgling number decreased (Ahola et al., 2009).

Temporal fluctuations of environmental conditions that affect fitness‐related traits are often correlated, or synchronized, over large distances (Liebhold et al., 2004). This environmental synchrony may, in turn, induce correlated temporal fluctuations in population abundance among spatially distinct populations, a phenomenon known as spatial population synchrony (Hansen et al., 2020; Liebhold et al., 2004). Spatial population synchrony often spans large spatial scales, with a general pattern of high synchrony among nearby populations and lower synchrony among more distant populations (Liebhold et al., 2004). Elton (1924) and Moran (1953) were the first to attribute spatial synchrony in population fluctuations to spatial correlation in environmental conditions. Since then, studies on a wide range of taxa, including birds, have shown that spatial synchrony in environmental conditions contributes to spatial synchrony in population abundance (Hansen et al., 2020; Koenig & Liebhold, 2016; Paradis et al., 1999; Sæther et al., 2007). Spatial synchrony in environmental conditions can also influence spatial synchrony in vital rates, like survival (Olmos et al., 2020), and fitness‐related traits, like body mass (Herfindal et al., 2020). Yet, despite numerous studies on geographical and temporal patterns of timing of breeding, clutch size, and fledgling number, as well as climatic effects on these traits (Both et al., 2004; Samplonius et al., 2018; Skagen & Adams, 2012), little is known about large‐scale synchrony in their trait values (but see Olin et al., 2020). Likewise, there is a lack of understanding of how spatial synchrony in traits is influenced by environmental conditions or scales up to spatial synchrony in population abundances. Laying date, clutch size, and fledgling number are the focus of some of the most extensive long‐term individual‐based studies, with multiple decades of data collected over multiple continents (Culina et al., 2021), making them ideal for studying spatial synchrony.

Here, we use a unique collection of data from 86 long‐term (i.e., at least 9 years) monitored populations of blue tits (*Cyanistes caeruleus*), great tits (*Parus major*), and pied flycatchers (*Ficedula hypoleuca*) at 44 different study sites across Europe to study spatial synchrony in trait values across populations. We focus on three fitness‐related traits (i.e., laying date, clutch size, and fledgling number) and quantify how spatial correlation in the temporal variation of trait values changes with distance between populations. We then examine the extent to which these spatial synchrony patterns can be explained by two local climatic variables: temperature and precipitation during spring. We expect that traits tightly linked to the environment, such as laying date, may show high correlations between trait values over large geographic areas.

## METHODS

### Study sites and data collection

**FIGURE 1:**
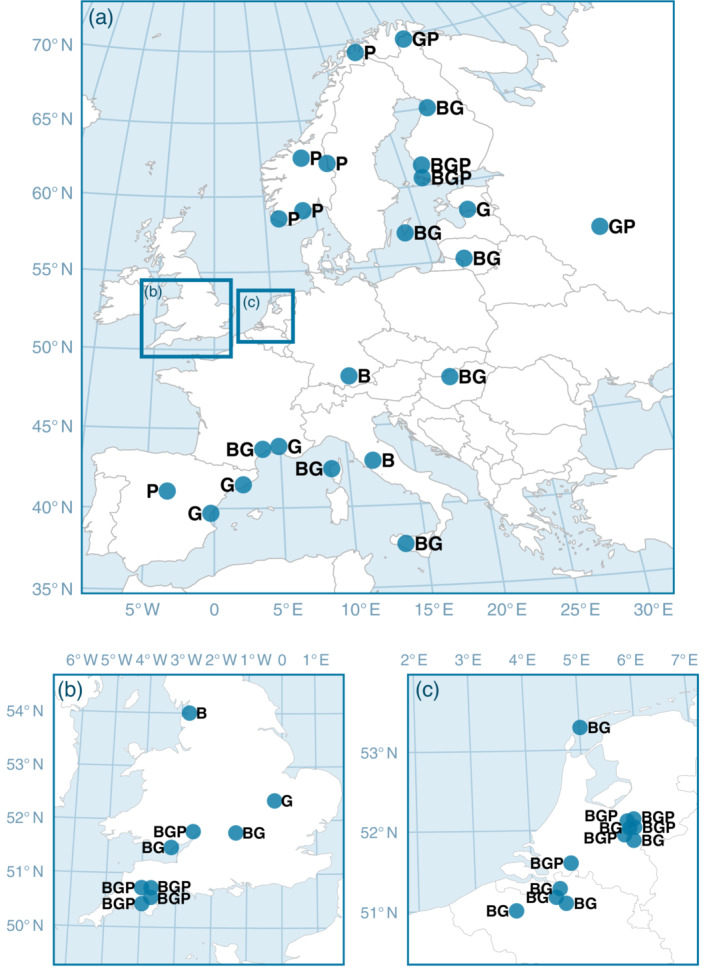
Map of the 86 studied populations of blue tit (B), great tit (G), and pied flycatcher (P) at 44 locations in (a) Europe, with insets of the (b) United Kingdom and (c) the Netherlands and Belgium.

Blue tits, great tits, and pied flycatchers are small passerines that breed in natural cavities and artificial nest boxes across Europe. Blue tits and great tits are mostly year‐round residents or partial migrants (Smallegange et al., 2010), whereas pied flycatchers are obligate migrants that travel to West Africa in fall and return in spring. We collated data from 86 populations (blue tit: *n* = 31, great tit: *n* = 35, pied flycatcher: *n* = 20) that had been monitored for at least 9 years. These populations came from nest box schemes at 44 locations in Europe (Figure 1). The studied populations occupied various woodland habitats dominated by deciduous, evergreen, or mixed forests. They ranged latitudinally from Sicily, Italy (37°35′ N) to Kevo, Finland (69°45′ N) and longitudinally from Okehampton, UK (3°59′ W) to Zvenigorod, Russia (36°51′ E), representing a large part of each species' breeding range. Metadata of most populations are available through the Studies on Populations of Individuals Birds (SPI‐Birds; www.spi-birds.org; Culina et al., 2021). The general procedure of data collection involved regular visits to all nest boxes throughout the breeding season. Brood‐specific information on laying date (i.e., the day the first egg was laid, 1 = April 1; note that smaller values are earlier in the year), clutch size (i.e., number of eggs), and fledgling number (i.e., number of chicks 13–16 days after hatching) were collected. When nests were not visited on the day the first egg was laid, laying date was calculated assuming that one egg was laid per day. For all analyses we only included first clutches that were not subjected to any experiments that could have affected the viability of parents or chicks (e.g., clutch size manipulation), and for each species and site we only retained years with two broods or more. For analyses on fledgling number, we only included broods with at least one fledgling. This way, fledgling number is essentially influenced by parental effort because complete brood losses due to predation or other external causes are excluded. Laying dates were not available for great tits in Dendles Wood, and fledgling numbers were not available for blue tits in Rome and Upeglynis and great tits in Gotland and Upeglynis. Overall, the study period spanned from 1955 to 2019, collectively including a total of 2670 study years. We used 126,667 brood records for analyses of laying date, 123,763 for clutch size, and 97,481 for fledging number. For an overview of sample sizes per population, see Vriend et al. (2022).

### Climatic variables

Two local climatic variables were used in this study. Mean temperature and precipitation have been widely associated with variation in the traits studied here (e.g., Bailey et al., 2022; Bowers et al., 2016) and identified as drivers of spatial synchrony in other animal groups (e.g., Herfindal et al., 2020). Several studies have suggested that temperature extremes rather than averages drive climate change responses (Bailey & van de Pol, 2016). However, because maximum and mean temperatures correlated strongly across our study populations (Appendix S1: Figure S1), we used mean temperature in further analyses. We extracted daily mean temperatures (°C) and daily precipitation (mm) from the corresponding 0.1° × 0.1° grid cell in the E‐OBS gridded data set version 20.0e (Cornes et al., 2018). The E‐OBS data set did not include data for three of 44 (7%) study sites. For Askainen and Cambridgeshire, we used data from the nearest‐neighboring grid cells in the E‐OBS data set, which were, respectively, 6 and 2 km from the study sites. For Vlieland, an island population in the Netherlands, no neighboring grid cells were available. Temperature data were used from grid cells corresponding to the neighboring island of Texel (18 km from the study site), which strongly correlated (Pearson's *r* = 0.999) with data from a Royal Dutch Meteorological Institute (KNMI) weather station on Vlieland (available for 1996–2017). Precipitation data were extracted from another KNMI weather station on Vlieland (Oost‐Vlieland, 4 km away).

For both climatic variables, we calculated annual values as the mean in February–May (i.e., the period before and during breeding) because this period was the most crucial to the species and traits studied here (e.g., Both et al., 2004; Visser et al., 2006). This was further confirmed by other studies using climate window analyses (Bailey et al., 2022; Samplonius et al., 2018), a statistical approach that identifies and quantifies weather signals and their critical time window on trait values (van de Pol & Bailey, 2019).

In addition to the two local climatic variables, we used the NAO index as a climatic variable on a larger, regional scale. We extracted daily data on the NAO index from the Climate Prediction Center of the National Weather Service (www.cpc.ncep.noaa.gov). Analyses involving the NAO index are available in Appendix S2.

### Effects of climatic variables on temporal variation in fitness‐related trait values

For each year in each population, we calculated median laying date, mean clutch size, and mean fledgling number. Because annual distributions of laying dates are often right‐skewed, the median is a more appropriate measure of the central tendency than the arithmetic mean for this trait. Hereafter, descriptors (mean and median) of population values for the three traits are referred to as average trait values. We first explored time trends in these average values for each trait separately using linear mixed‐effects models of the form

(1) $$Y_{\mathrm{ijk}}=\beta_{\mathrm{int},j}+b_{\mathrm{int},\mathrm{jk}}+\left(\beta_{\text{year},j}+b_{\text{year},\mathrm{jk}}\right)X_{\mathrm{ijk}}+\varepsilon_{\mathrm{ijk}}$$

where $Y_{\mathrm{ijk}}$ are the average trait values per year i, species j, and location k, $\beta_{\mathrm{int},j}$ is a species‐specific intercept, $b_{\mathrm{int},\mathrm{jk}}$ denotes random intercepts for each species j at location k (i.e., population jk), assumed to have a normal prior distribution with mean 0 and standard deviation (SD) $\sigma_{b_{\mathrm{int}}}$, $\beta_{\text{year},j}$ is a species‐specific slope for the linear time trend, $b_{\text{year},\mathrm{jk}}$ denotes random slopes for the linear time trends for each population jk, assumed to have a normal prior distribution with mean 0 and SD $\sigma_{b_{\text{year}}}$, $X_{\mathrm{ijk}}$ are time indicators per population jk, and $\varepsilon_{\mathrm{ijk}}$ is a residual error, assumed to have a normal prior distribution with mean 0 and SD $\sigma_{\varepsilon }$.

In a second set of models, we explored the effects of local climatic variables (mean temperature and mean precipitation in February–May) on the average trait values. Climatic variables were normalized (i.e., subtracting the mean and dividing by the SD) to compare their relative effects on the average trait values. For each combination of trait and climatic variable, we ran a linear mixed‐effects model of the form

(2) $$\begin{array}{c} Y_{\mathrm{ijk}}=\beta_{\mathrm{int},j}+b_{\mathrm{int},\mathrm{jk}}+\left(\beta_{\text{year},j}+b_{\text{year},\mathrm{jk}}\right)X_{\mathrm{ijk}}\\ \;+\left(\beta_{\text{clim},j}+b_{\text{clim},\mathrm{jk}}\right)Z_{\mathrm{ijk}}+\varepsilon_{\mathrm{ijk}} \end{array}$$

where $\beta_{\text{clim},j}$ is a species‐specific slope for the climatic variable, $b_{\text{clim},\mathrm{jk}}$ denotes random slopes for the climatic variables for each population jk, assumed to have a normal prior distribution with mean 0 and SD $\sigma_{b_{\text{clim}}}$, $Z_{\mathrm{ijk}}$ are the normalized climatic variables per population jk, and the other parameters and variables are as defined in Equation (1).

Linear mixed‐effects models were run using *brms* version 2.15.0 (Bürkner, 2017) in R version 4.0.5 (R Core Team, 2021). We used default priors and ran four Markov chains for 2000 iterations with a burn‐in of 1000, resulting in 4000 posterior samples. Chain convergence was assessed using the convergence diagnostic $\hat{R}$ and the effective sample size (Vehtari et al., 2021).

### Effects of climatic variables on spatial synchrony in fitness‐related trait values

For the analysis of spatial synchrony, annual average trait values were linearly detrended (i.e., retaining residuals from a linear regression of average trait value against year) and normalized. By detrending and normalizing the average trait values, we explored spatial synchrony in the temporal fluctuations of the average trait values relative to long‐term population means rather than spatial synchrony in absolute population differences and shared common trends. Following Engen et al. (2005), we assumed a spatial autocorrelation function for each species–trait combination of the form

(3) $$\begin{array}{c} \rho \left(d\right)=\rho_{\infty }+\left(\rho_{0}-\rho_{\infty }\right)e^{-d^{2}/2l^{2}}\; \end{array}$$

where $\rho_{0}$ and $\rho_{\infty }$ are the correlation of average trait values as distance approaches zero and infinity, respectively, $e^{-d^{2}/2l^{2}}$ is a Gaussian positive‐definite autocorrelation function where d is the distance between populations (in kilometers), and the standard deviation l (in kilometers) is a standardized measure of the scale of spatial autocorrelation (Engen et al., 2005; Lande et al., 1999). The spatial scale l can be considered the characteristic distance at which the temporal fluctuations of an ecological property (here trait values) remain correlated or, in other words, the size of the region over which temporal fluctuations are synchronized (Jarillo et al., 2018).

There are various approaches to quantifying spatial synchrony, which can be categorized into parametric models, like the one we used here, and nonparametric models (e.g., Koenig & Liebhold, 2016). Contrary to nonparametric approaches, our model (Equation 3) assumed that the spatial autocorrelation structure was Gaussian. As a result, the model parameters ($\rho_{0}$, $\rho_{\infty }$, l) were assumed to be positive, although the correlation between two sites might be negative, especially at large distances. The advantage of our parametric approach is that the model parameters have a biological interpretation that allows the user to formally compare spatial synchrony across traits or species and assess the effects of potential drivers of spatial synchrony.

The observations of the detrended and normalized average trait value $\tilde{Y}$ of all locations in each year were assumed to follow a multivariate normal distribution, ${\tilde{Y}}_{t}\sim \mathrm{MVN}\left(0,\sum_{t}\right)$. The off‐diagonal elements of the variance–covariance matrix ∑ were defined by $\rho_{0}$, $\rho_{\infty }$, and l (Equation 3) given distance d, and the diagonal elements were set to 1. Since data from different locations were available over different but partly overlapping periods, the set of locations varied among years. Generally, the more a pair of time series overlapped, the larger the contribution to the likelihood. The total log‐likelihood was the sum of annual log‐likelihoods and optimized numerically to provide estimates for $\rho_{0}$, $\rho_{\infty }$, and l. Distributions of these parameters were obtained by a parametric bootstrapping procedure involving data simulation from the multivariate normal distribution as defined earlier and based on the estimated parameters and the annual sets of locations included in the observed data (Engen et al., 2005). This procedure was undertaken 2000 times, resulting in 2000 bootstrap replicates. The multivariate normal distribution was obtained from *mvtnorm* version 1.1‐1 (Genz et al., 2020) in R version 4.0.5 (R Core Team, 2021).

In addition to laying date, clutch size, and fledgling number, we quantified spatial synchrony in fledgling success (i.e., proportion fledged, calculated as fledgling number/clutch size) because the constraint of clutch size on fledgling number might confound the spatial synchrony patterns in fledgling number. Spatial synchrony patterns in fledgling number and fledgling success were similar for all three species (see Appendix S3).

Finally, we examined the extent to which the climatic variables (mean temperature and mean precipitation) contributed to spatial synchrony in average trait values. Following Grøtan et al. (2005) and Sæther et al. (2007), we regressed the population‐specific annual average trait values against population‐specific annual means of the climatic variables using separate linear regression models for each combination of species, trait, and climatic variable. The residuals were normalized and used in the spatial autocorrelation model (Equation 3; three species, three traits, two climatic variables, 18 models in total) to calculate the spatial synchrony in trait values after accounting for the effect of climatic variables.

Populations at the southern edges of the species' distribution ranges generally experience warmer temperatures and are more likely to face extreme temperatures. Despite that, the most southern populations of all three study species did not disproportionately influence the findings of this study since results were similar when considering a subset of populations located at higher latitudes (>45° N; see Appendix S4).

## RESULTS

### Temporal variation in fitness‐related trait values

**FIGURE 2:**
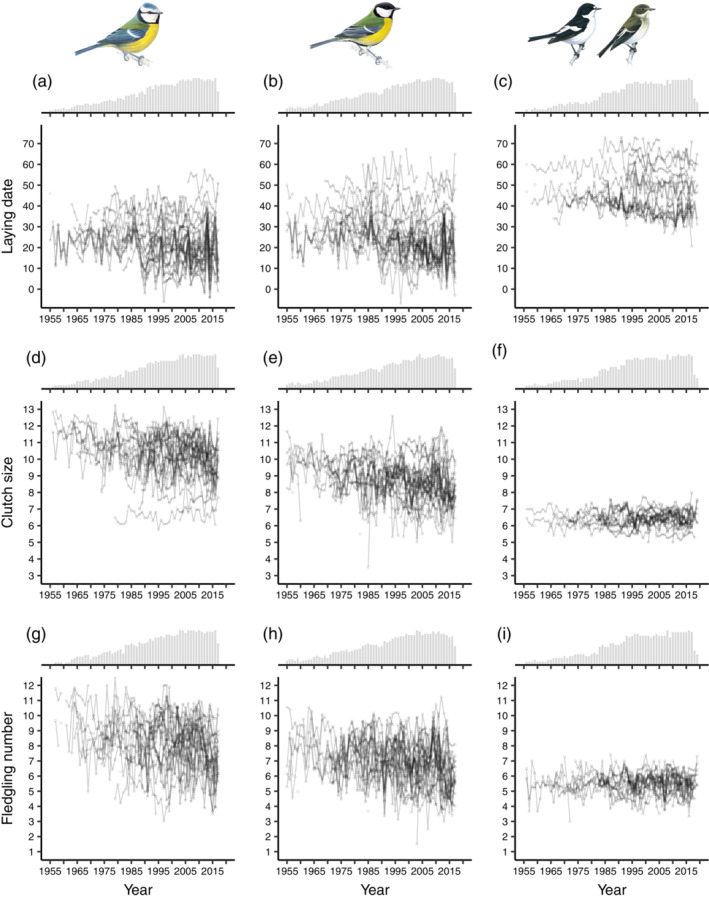
Temporal variation in (a–c) laying dates (1 = April 1), (d–f) clutch size, and (g–i) fledgling number of blue tit (a, d, g), great tit (b, e, h), and pied flycatcher (c, f, i) populations. Lines and points correspond to population time series of annual average trait values (median laying dates, mean clutch sizes, and mean fledgling numbers), allowing years with missing data. Histograms show annual data density, that is, the relative frequency of populations available per year. The analysis of spatial synchrony was carried out over the detrended and normalized annual average trait values (Appendix S1: Figure S5). Bird drawings reproduced with permission of Mike Langman, RSPB (rspb‐images.com).

The timing of laying advanced over time for all species (posterior mode [95% credible interval]: blue tit: −0.175 [−0.214, −0.133]; great tit: −0.168 [−0.207, −0.131]; pied flycatcher: −0.165 [−0.211, −0.117] in days per year), but the strength of this trend differed between populations ($\sigma_{b_{\text{year}}}$: 0.059 [0.032, 0.090]; Appendix S1: Table S1 and Figure S2). Annual median laying dates occurred within a two‐month period for all species (Figure 2a–c) but were earlier for resident blue tits (range: 26 March–28 May, mode: 22 April, *n* = 898) and great tits (25 March–7 June, 23 April, *n* = 1041) than for migratory pied flycatchers (21 April–13 June, 10 May, *n* = 662).

Clutch size showed a trend toward smaller clutches over time for blue tits (−0.021 [−0.026, −0.015] eggs per year) and great tits (−0.017 [−0.022, −0.012] eggs per year), with varying strengths among populations ($\sigma_{b_{\text{year}}}$: 0.008 [0.006, 0.012]), but not for pied flycatchers (0.005 [−0.002, 0.011] eggs per year; Appendix S1: Table S1 and Figure S3). Annual mean clutch size varied strongly over time for blue tits (CV = 0.137, *n* = 899) and great tits (CV = 0.141, *n* = 1046), but less for pied flycatchers (CV = 0.075, *n* = 670; Figure 2d–f).

Similarly, annual mean fledgling number showed a trend toward fewer fledglings over time for blue tits (−0.019 [−0.028, −0.011] fledglings per year) and great tits (−0.018 [−0.025, −0.010] fledglings per year), with varying strengths among populations ($\sigma_{b_{\text{year}}}$: 0.013 [0.010, 0.018]), but not for pied flycatchers (0.000 [−0.009, 0.009] fledglings per year; Appendix S1: Table S1 and Figure S4). Annual mean fledgling number varied strongly over time for blue tits (CV = 0.209, *n* = 845) and great tits (CV = 0.211, *n* = 1020), and less for pied flycatchers (CV = 0.125, *n* = 657; Figure 2g–i).

### Effects of climatic variables on fitness‐related trait values

We observed earlier laying with increasing temperatures for all three species, but the effect was stronger for blue tits and great tits compared to pied flycatchers (Table 1). The effects of mean temperature showed large variation among populations. We observed weak effects of mean temperature on clutch size; clutch size increased with mean temperatures for great tits and pied flycatchers but not for blue tits. In addition, we observed no overall effect of mean temperature on fledgling numbers for any of the species, but there was large variation among populations (Table 1).

**TABLE 1:** Effects of climatic variables (mean temperature and mean precipitation in February–May) on laying date, clutch size, and fledgling number for blue tits (B), great tits (G), and pied flycatchers (P).

Trait		Temperature		Precipitation	
	Parameter	Mode	95% CrI	Mode	95% CrI
Laying date	$\beta_{\text{clim},B}$	−11.716	−13.390 to −10.218	0.749	0.149 to 1.182
	$\beta_{\text{clim},G}$	−10.558	−11.977 to −8.921	0.822	0.330 to 1.328
	$\beta_{\text{clim},P}$	−5.614	−7.629 to −3.819	0.071	−0.468 to 0.609
	$\sigma_{b_{\text{clim}}}$	3.391	2.627 to 4.488	0.060	0.009 to 0.567
Clutch size	$\beta_{\text{clim},B}$	0.158	−0.012 to 0.293	−0.005	−0.066 to 0.068
	$\beta_{\text{clim},G}$	0.155	0.028 to 0.287	−0.008	−0.079 to 0.048
	$\beta_{\text{clim},P}$	0.225	0.096 to 0.406	−0.029	−0.100 to 0.042
	$\sigma_{b_{\text{clim}}}$	0.021	0.003 to 0.189	0.014	0.002 to 0.092
Fledgling number	$\beta_{\text{clim},B}$	0.148	−0.093 to 0.423	0.026	−0.077 to 0.126
	$\beta_{\text{clim},G}$	0.209	−0.014 to 0.449	−0.048	−0.149 to 0.040
	$\beta_{\text{clim},P}$	−0.062	−0.317 to 0.248	−0.045	−0.491 to 0.058
	$\sigma_{b_{\text{clim}}}$	0.349	0.136 to 0.536	0.014	0.001 to 0.120

*Note*: Effects were estimated using linear mixed‐effects models (Equation 2), where $\beta_{\text{clim},j}$ is the slope of the climatic variable per species *j* and $\sigma_{b_{\text{clim}}}$ the standard deviation (SD) of the normal distribution from which random slopes for the climatic variables for each species j at location k were drawn. $\beta_{\text{clim},j}$ for laying date are given in days per SD of the climate variable [i.e., temperature (°C) or precipitation (mm)], for clutch size in eggs per SD of the climate variable, and for fledgling number in fledglings per SD of the climate variable. The analyses were based on 2601 observations (years) from 85 populations for laying date, 2615 observations from 86 populations for clutch size, and 2522 observations from 82 populations for fledgling number. Estimates are given by the posterior mode and 95% credible interval (95% CrI). Full model outputs can be found in Appendix S1: Tables S2 and S3.

The effects of mean precipitation were toward later laying with increasing precipitation for blue tits and great tits, but we found no evidence for such an effect for pied flycatchers. In addition, we found no evidence for an effect of mean precipitation on clutch sizes and fledgling numbers in any of the species (Table 1). In general, for each trait, the effects of mean temperature were stronger and more variable than the effects of mean precipitation. Temporal variation in climatic variables is shown in Appendix S1: Figure S6.

### Spatial synchrony in fitness‐related trait values

**FIGURE 3:**
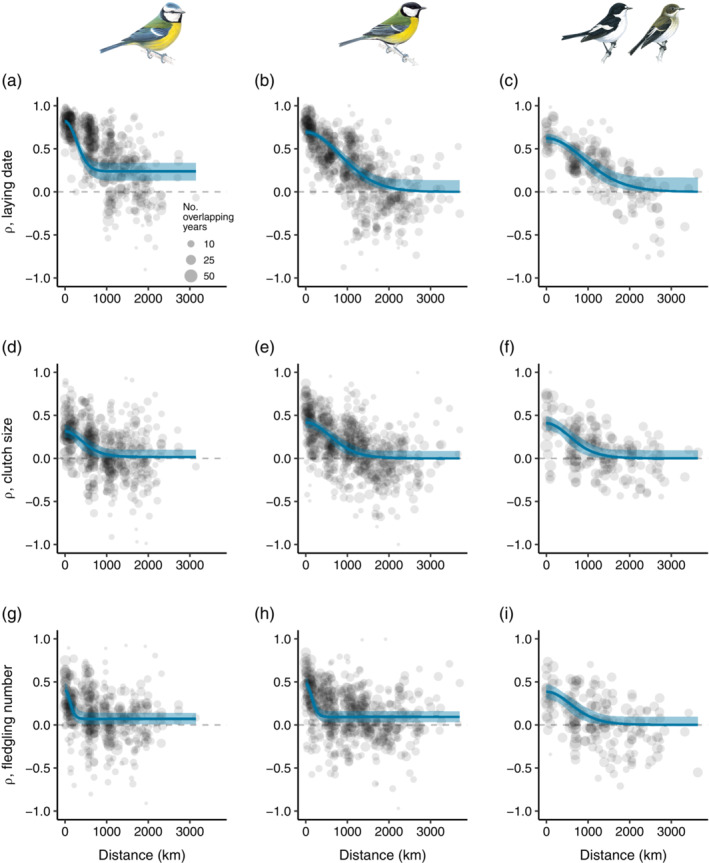
Spatial synchrony in (a–c) laying date, (d–f) clutch size, and (g–i) fledgling number of blue tit (a, d, g), great tit (b, e, h), and pied flycatcher (c, f, i) populations in relation to the distance (in kilometers) between populations. Blue solid lines are the median and blue ribbons the 95% confidence interval based on 2000 bootstrap replicates. Gray points are correlations between the time series of pairs of sites whose size is proportional to the number of overlapping years between them. Spatial synchrony parameters (${\hat{\rho }}_{0}$, ${\hat{\rho }}_{\infty }$, and $\hat{l}$) were restricted to positive values. Bird drawings reproduced with permission of Mike Langman, RSPB (rspb‐images.com).

For each species–trait combination, spatial synchrony decreased with increasing distance between populations (Figure 3). Estimates of the correlation at zero distance (${\hat{\rho }}_{0}$) were high for laying date (median ${\hat{\rho }}_{0}$ range: 0.624–0.800) and lower for clutch size and fledgling number in all species (median ${\hat{\rho }}_{0}$ range: 0.314–0.477; Table 2, Appendix S1: Figure S7a–c). Estimates of the correlation at infinity (${\hat{\rho }}_{\infty }$) approached zero for most species–trait combinations (Table 2, Appendix S1: Figure S7d–f), except for laying date in blue tits and fledgling number in blue tits and great tits.

**TABLE 2:** Estimates of spatial synchrony parameters, correlation at zero distance ${\hat{\rho }}_{0}$, correlation at infinity ${\hat{\rho }}_{\infty }$, and spatial scale $\hat{l}$ (in kilometers, i.e., the characteristic distance at which the temporal fluctuations of trait values remain correlated) for laying date, clutch size, and fledgling number in blue tits, great tits, and pied flycatchers.

Parameter		Blue tit		Great tit		Pied flycatcher	
	Trait	Median	95% CI	Median	95% CI	Median	95% CI
${\hat{\rho }}_{0}$	LD	0.800	0.769–0.828	0.657	0.619–0.694	0.624	0.564–0.679
	CS	0.314	0.243–0.389	0.418	0.355–0.476	0.410	0.330–0.488
	FN	0.400	0.308–0.484	0.477	0.402–0.547	0.385	0.298–0.468
${\hat{\rho }}_{\infty }$	LD	0.263	0.163–0.355	0.000	0.000–0.140	0.000	0.000–0.139
	CS	0.017	0.000–0.100	0.000	0.000–0.086	0.000	0.000–0.094
	FN	0.070	0.008–0.140	0.094	0.033–0.158	0.000	0.000–0.096
$\hat{l}$	LD	247	204–305	841	710–997	734	585–898
	CS	422	225–625	595	447–767	565	385–769
	FN	119	73.8–199	141	101–199	596	386–825

*Note*: Median and 95% confidence interval are based on 2000 bootstrap replicates. Spatial synchrony parameters were restricted to be positive.

Abbreviations: CI, confidence interval; CS, clutch size; FN, fledgling number; LD, laying date.

Estimates of the scale of spatial autocorrelation ($\hat{l}$ in kilometers, i.e., the characteristic distance at which the temporal fluctuations of trait values remain correlated) were high for laying date and clutch size in great tits and pied flycatchers (median $\hat{l}$ range: 565–841 km) but relatively low in blue tits (median $\hat{l}$ range: 247–422 km, Table 2, Appendix S1: Figure S7g–i). The scale of spatial autocorrelation for fledgling number was substantially lower than for the other traits in blue tits and great tits (median $\hat{l}$ range: 119–141 km), but not in pied flycatchers.

### Effect of climatic variables on spatial synchrony

**FIGURE 4:**
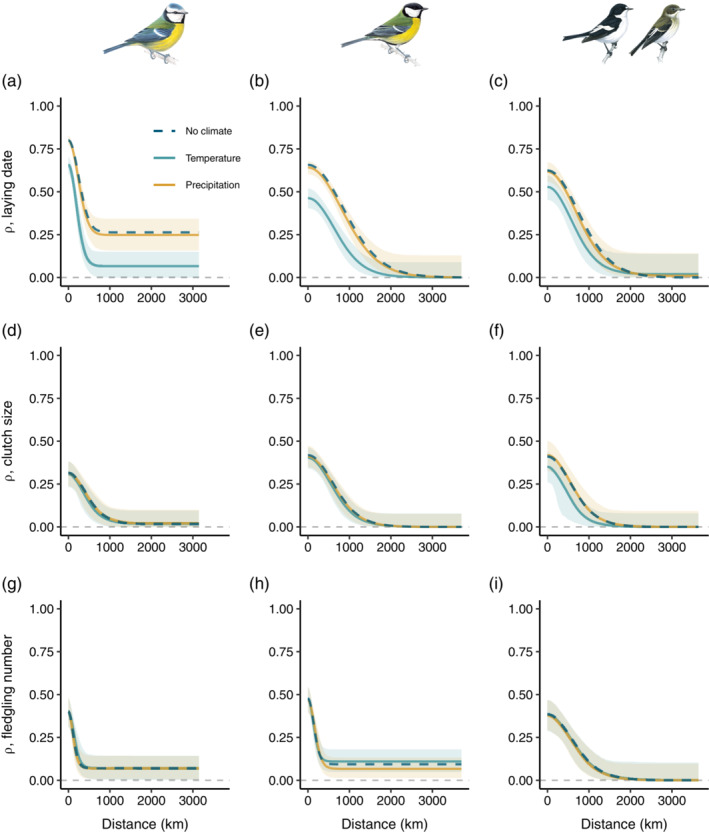
Spatial synchrony in (a–c) laying date, (d–f) clutch size, and (g–i) fledgling number of blue tit (a, d, g), great tit (b, e, h), and pied flycatcher (c, f, i) populations in relation to the distance (in kilometers) between populations. Blue dashed lines are the spatial synchrony in the traits without accounting for the effects of climatic variables (Figure 3), teal lines are the spatial synchrony in the residuals after accounting for the effects of mean temperature in February–May, and yellow lines are the spatial synchrony in the residuals after accounting for the effects of mean precipitation in February–May. Lines are the median and ribbons the 95% confidence interval based on 2000 bootstrap replicates. Spatial synchrony parameters (${\hat{\rho }}_{0}$, ${\hat{\rho }}_{\infty }$, and $\hat{l}$) were restricted to positive values. Bird drawings reproduced with permission of Mike Langman, RSPB (rspb‐images.com).

Accounting for variation in mean temperature substantially decreased the spatial synchrony in laying date at both short and longer distances in blue tits and great tits, whereas spatial synchrony remained mostly unchanged for pied flycatchers (Figure 4a–c, Appendix S1: Figure S8). In contrast, we found no contribution of mean temperature to the spatial synchrony in clutch size (Figure 4d,e, Appendix S1: Figure S8) or fledgling number (Figure 4g–i, Appendix S1: Figure S8), except for a small contribution of mean temperature to clutch size in pied flycatchers (Figure 4f, Appendix S1: Figure S8). We found no evidence for synchronizing effects of mean precipitation for any species‐trait combination (Figure 4, Appendix S1: Figure S9).

## DISCUSSION

Using 86 long‐term monitored populations of three common European hole‐nesting passerines from 44 different study sites, we found a high degree of spatial synchrony in laying date (Figure 3a–c) and a lower degree in clutch size and fledgling number (Figure 3d–i), a pattern that was consistent across species. We also found a strong effect of mean temperature on temporal variation in trait values within populations (Table 1) and on spatial synchrony among populations for laying date, particularly in blue tits and great tits (Figure 4a,b).

### Effects of temperature on laying dates and their spatial synchrony

Seasonal timing of breeding has strong fitness consequences for all three species studied here (Perrins, 1970). Reproductive success often decreases during the breeding season (Perrins & McCleery, 1989), but breeding too early can also be costly (Bowers et al., 2016). In temperate regions, strong seasonality in the environment leads to a short optimal breeding period in terms of energy and nutrient availability (Perrins, 1970) that varies in timing and length among years (Marrot et al., 2018). In response to warming springs, many bird populations have advanced their timing of breeding (Both et al., 2004; Hällfors et al., 2020). Other populations show no such trend (Keogan et al., 2018; Vatka et al., 2014), sometimes resulting in a phenological mismatch between food abundance and nestlings' nutritional needs (Visser et al., 1998). Even if birds advance their breeding time, a phenological mismatch can still occur when the phenology of food supplies advances at a different rate than the birds' breeding phenology (Mayor et al., 2017). Ultimately, we can expect that between‐year variation in the timing of breeding is explained by between‐year variation in the environment (Visser et al., 2010). Here, we found evidence for strong effects of mean local temperature in February–May on laying dates and spatial synchrony in laying date, particularly in blue tit and great tit populations and at large distances. A previous study also demonstrated a synchronizing effect of temperature on the population abundance of blue tits and great tits in Central Europe (Sæther et al., 2007), and our results confirmed that large‐scale variation in laying date could be attributed to spatial covariation in temperature (Visser et al., 2003).

In contrast to the synchronizing effects of mean local temperature on blue tit and great tit laying dates, we found that mean temperature contributed less to spatial synchrony in pied flycatcher laying dates. The time window used for our analysis (i.e., February–May) overlaps largely with the timing of pied flycatcher spring migration. Long‐distance migrants, like pied flycatchers, experience a greater range of challenges across their annual cycle (Rushing et al., 2017). Their timing of breeding is constrained by the timing of spring arrival, which in turn is affected by the conditions they experience before and during migration (Saino et al., 2011), including temperature and precipitation throughout their migration trajectory (Ahola et al., 2004; Saino et al., 2007). Because there is large variation in how conditions across the annual cycle may have changed (Ahola et al., 2004), populations of migratory birds differ substantially in their response to abiotic factors at the breeding grounds (Both & te Marvelde, 2007). Additionally, the timing of breeding of migrants may be influenced by competition with earlier breeding resident species (Samplonius et al., 2018), leading migrants to adjust their breeding time based on the residents' breeding time (Samplonius & Both, 2017).

### Effects of precipitation on trait values and their spatial synchrony

We found little evidence for the effect of mean precipitation on spatial synchrony in any species–trait combination. Spatial synchrony in precipitation is generally lower than in temperature (Herfindal et al., 2020; Koenig & Liebhold, 2016), which could explain why we found no effect of precipitation on spatial synchrony in this study. Yet, even when precipitation shows spatial synchrony, this may lead to similar spatial synchrony in species' trait values. Variation in precipitation patterns can affect breeding time and reproductive success of small passerines (Bowers et al., 2016). These effects can occur indirectly through reduced food availability or directly through increased energy expenditure (Radford et al., 2001), both of which can have negative consequences for nestling growth and survival (Öberg et al., 2015; Radford et al., 2001). However, precipitation has also been positively associated with nestling mass and growth in other studies (Eeva et al., 2020). The contradictory results in the literature may indicate that the effects of precipitation can vary substantially between individuals and populations. As such, geographically close populations may respond differently to changes in climatic variables (Bonamour et al., 2019; Sæther et al., 2003).

### Effects of other drivers on trait values and their spatial synchrony

We found evidence for large annual variation in clutch size and fledgling number for the resident blue tits and great tits, with smaller clutches and fewer fledglings over time. This pattern was not observed for the migratory pied flycatchers for which clutch size and fledgling number remained constant over time and buffered against environmental variation. For all three species, unlike laying date, we found no evidence for the effects of mean temperature or mean precipitation in February–May on spatial synchrony in clutch size and fledgling number. Spatial synchrony in clutch size and fledgling number generally acted at a smaller spatial scale than spatial synchrony in laying date. Furthermore, after accounting for mean temperature, the spatial correlation in laying dates remained high at shorter distances. This implies that more local factors play an important role in driving the fluctuations in the values of the traits studied here. Spatial autocorrelation in a variety of factors may generate smaller‐scale spatial synchrony in laying date, clutch size, and fledgling number. First, hole‐nesting passerines breed in a variety of habitats with varying quality (e.g., Blondel et al., 1993). Spatial structuring of habitats of different quality may cause spatial covariation in clutch size and breeding performance (Lambrechts et al., 2004).

Second, habitat heterogeneity may influence spatial synchrony through density‐dependent effects on breeding parameters like clutch size (Dhondt et al., 1992). Because locations with different density‐dependent dynamics are expected to show reduced spatial synchrony (Walter et al., 2017), the spatial scale of density dependence determines the spatial scale of trait synchrony. In general, the spatial scale of synchrony in population abundances (Kendall et al., 2000; Lande et al., 1999) and the spatial covariation in phenotypic selection (Engen & Sæther, 2016) decrease with increasing strength of density dependence. Further, population density may also affect the relationship between environmental variables, such as spring temperature, and traits, such as laying date and clutch size (Møller et al., 2020).

Third, in the case of tits, individuals are facultative multiple breeders in some parts of the species range (Verhulst et al., 1997). Pairs producing multiple clutches must optimize their fitness over multiple clutches, which affects the breeding time, clutch size, and fledgling success of the first brood (Verhulst et al., 1997). The incidence of double brooding in tits varies geographically, annually, and between habitat types (Husby et al., 2009). If populations in similar habitats show similar temporal dynamics of the incidence of double brooding, habitat heterogeneity and local density dependence may then synchronize the dynamics of clutch size and fledgling number of first clutches.

Fourth, for tits in temperate regions, beech mast forms a major food source in winter (Perdeck et al., 2000; Perrins, 1965). In great tits, beech mast variation has been linked to increased survival (Perdeck et al., 2000) and recruitment (Grøtan et al., 2009). Temporal dynamics of beech mast tend to be consistent over large distances (Perrins, 1965), inducing spatial synchrony in abundance (Sæther et al., 2007), which may indirectly generate spatial synchrony in tit fitness‐related traits. If beech mast plays a role in the spatial synchrony in traits, it is likely restricted in time and space because annual variation in beech seed production has decreased recently (Bogdziewicz et al., 2020), and great tits in evergreen forests and blue tits, in general, rely on other food sources (e.g., supplemental feeding; Orell, 1989).

Besides synchronous environmental fluctuations, movement between spatially distinct populations has also been identified as a driver of spatial population synchrony (Lande et al., 1999), particularly on local scales (Paradis et al., 1999). Median natal dispersal distances in these species are typically short (Chernetsov et al., 2006; Paradis et al., 1998; Tufto et al., 2005; van Balen & Hage, 1989), and median breeding dispersal distances are even shorter (Eeva et al., 2008; Paradis et al., 1998, 2002; Thomson et al., 2003), despite the fact that some individuals may disperse up to hundreds of kilometers to suitable breeding sites, especially when local population densities are high (Both et al., 2012; Matthysen, 2005; Paradis et al., 2002). Therefore, the spatial scale of dispersal between populations (Paradis et al., 1998, 2002; Tufto et al., 2005) is likely too short to induce the synchronous fluctuations in fitness‐related trait values reported here.

### Implications of spatial synchrony in fitness‐related trait values

Spatial synchrony in population abundance often spans large spatial scales, with a general pattern of high correlation between nearby populations and lower correlation when the distance between populations increases (Koenig, 2002; Liebhold et al., 2004). Here, we showed that spatial synchrony in fitness‐related trait values could act over similarly large distances. In fact, except for fledgling number in blue tits and great tits, the spatial scales of synchrony in this study were larger than for spatial synchrony in abundances of blue tit (mean $\hat{l}$ = 380 km) and great tit (mean $\hat{l}$ = 34 km) populations in Europe (Sæther et al., 2007). Because our results were consistent across species and traits, large‐scale spatial synchrony in trait values is likely for similar species and traits. Fitness‐related traits that show consistent responses to specific environmental variables, like laying date does to temperature, are likely candidates to have synchronous dynamics.

Climate change and other environmental perturbations may increase spatial population synchrony. For example, in a study on 49 widespread North American wintering bird species, spatial synchrony in population abundance increased over a period of 50 years, in parallel to an increase of spatial synchrony in temperature (Koenig & Liebhold, 2016). As a result of increased spatial synchrony, the probability of correlated declines in population abundances may increase, increasing the risk of species extinction (Heino et al., 1997; Pearson et al., 2014). Spatiotemporal fluctuations in vital rates or population abundances may be impacted not only directly by synchronized fluctuations in environmental conditions (i.e., environmental synchrony) but also indirectly by environment‐induced spatial synchrony in fitness‐related trait values. Future studies should therefore aim to understand under what conditions spatial synchrony in fitness‐related trait values can help explain spatiotemporal fluctuations in vital rates or population abundances and quantify the relative contributions of spatial trait synchrony in relation to other drivers of spatial population synchrony, such as movement and the environment. In the current context of global change and biodiversity loss, it will be especially valuable to explore the use of spatial trait synchrony as an indicator of spatial population synchrony, which could ultimately affect the risk of extinction.

## AUTHOR CONTRIBUTIONS

Stefan J. G. Vriend, Vidar Grøtan, Marlène Gamelon, and Bernt‐Erik Sæther conceived the study. All others provided data. Stefan J. G. Vriend and Liam D. Bailey compiled the data set. Stefan J. G. Vriend conducted the analyses with advice from Vidar Grøtan, Marlène Gamelon, and Bernt‐Erik Sæther. Stefan J. G. Vriend wrote the first draft of the manuscript. All others provided feedback on later drafts of the manuscript.

## CONFLICT OF INTEREST

The authors declare no conflict of interest.

## Supporting information


Appendix S1

Appendix S2

Appendix S3

Appendix S4

## Data Availability

The data supporting the results and the R code for the analyses (Vriend et al., 2022) are available in Figshare at https://doi.org/10.6084/m9.figshare.14972259. Daily North Atlantic Oscillation index values were downloaded from the National Weather Service Climate Prediction Center (www.cpc.ncep.noaa.gov), as described in Appendix S2.
