# Supplementary material for: Temperature synchronizes temporal variation in laying dates across European hole‐nesting passerines
Source: Ecology. 2022 Dec 21;104(2):e3908. doi: 10.1002/ecy.3908 (PMC10078612; doi:10.1002/ecy.3908)
Supplement: Supplementary file 1 — Appendix S1 [file ECY-104-0-s004.pdf]

Supplementary Materials to

Temperature synchronizes temporal variation in laying dates across European hole-nesting passerines

Ecology

Stefan J.G. Vriend, Vidar Grøtan, Marlène Gamelon, Frank Adriaensen, Markus P. Ahola, Elena Álvarez, Liam D. Bailey, Emilio Barba, Jean-Charles Bouvier, Malcolm D. Burgess, Andrey Bushuev, Carlos Camacho, David Canal, Anne Charmantier, Ella F. Cole, Camillo Cusimano, Blandine F. Doligez, Szymon M. Drobniak, Anna Dubiec, Marcel Eens, Tapio Eeva, Kjell Einar Erikstad, Peter N. Ferns, Anne E. Goodenough, Ian R. Hartley, Shelley A. Hinsley, Elena Ivankina, Rimvydas Juškaitis, Bart Kempenaers, Anvar B. Kerimov, John Atle Kålås, Claire Lavigne, Agu Leivits, Mark C. Mainwaring, Jesús Martínez-Padilla, Erik Matthysen, Kees van Oers, Markku Orell, Rianne Pinxten, Tone Kristin Reiertsen, Seppo Rytkönen, Juan Carlos Senar, Ben C. Sheldon, Alberto Sorace, János Török, Emma Vatka, Marcel E. Visser, Bernt-Erik Sæther

Appendix S1: Additional figures and tables

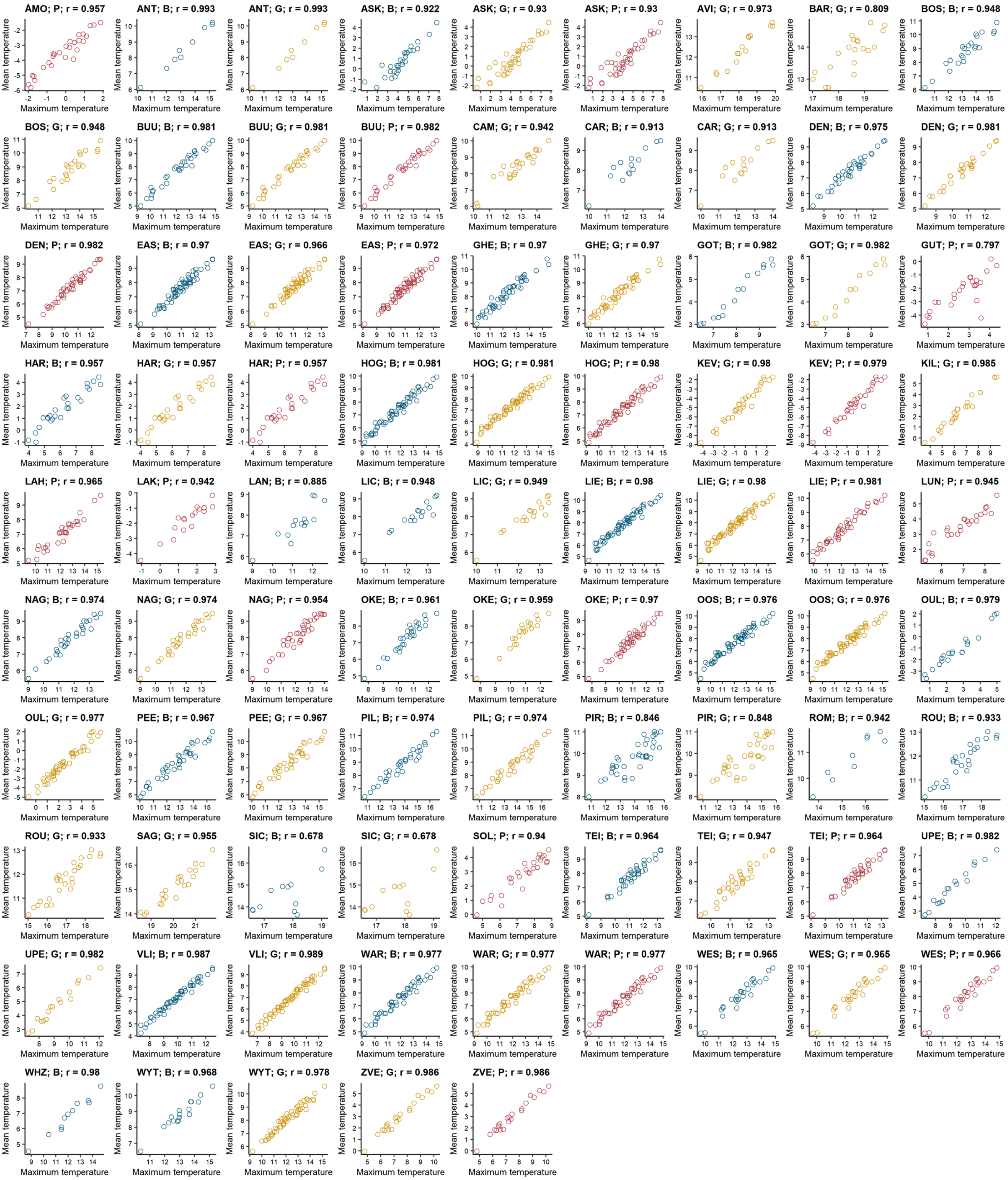

Figure S1. Comparison between mean and maximum temperatures for blue tit (B, blue), great tit (G, yellow), and pied flycatcher (P, red) populations using Pearson's correlation coefficient (r). Points correspond to annual means calculated from daily values in February-May, allowing years with missing data. Three-letter codes are location identifiers.

Table S1. Outputs of linear mixed-effects models of time trends in annual average trait values of median laying date (LD), mean clutch size (CS), and mean fledgling number (FN) for blue tits (B), great tits (G), and pied flycatchers (P). Models included an intercept per species ($\beta_{\text{int},j}$), a slope for the linear time trend per species ($\beta_{\text{year},j}$), random intercepts per population ($b_{\text{int},jk}$) and random slopes of the linear time trends per population ($b_{\text{year},jk}$). The analyses were based on 2,601 observations (years) from 85 populations for laying date, 2,615 observations from 86 populations for clutch size, and 2,522 observations from 82 populations for fledgling number. Estimates are given by the posterior mode and 95% credible interval (95% CrI).

Trait	Parameter		Mode	95% CrI
LD	Intercept	$\beta_{\text{int},B}$	31.445	27.185 – 35.518
		$\beta_{\text{int},G}$	33.500	29.502 – 37.131
		$\beta_{\text{int},P}$	54.410	49.309 – 59.491
	Year *(in days per year)*	$\beta_{\text{year},B}$	-0.175	-0.214 – -0.133
		$\beta_{\text{year},G}$	-0.168	-0.207 – -0.131
		$\beta_{\text{year},P}$	-0.165	-0.211 – -0.117
	SD random intercepts	$\sigma_{b_{\text{int}}}$	10.492	9.022 – 12.565
	SD random slopes	$\sigma_{b_{\text{year}}}$	0.059	0.032 – 0.090
	SD residual	σ_{ε}	5.045	4.915 – 5.192
CS	Intercept	$\beta_{\text{int},B}$	10.980	10.567 – 11.366
		$\beta_{\text{int},G}$	9.261	8.897 – 9.638
		$\beta_{\text{int},P}$	6.113	5.711 – 6.639
	Year *(in eggs per year)*	$\beta_{\text{year},B}$	-0.021	-0.026 – -0.015
		$\beta_{\text{year},G}$	-0.017	-0.022 – -0.012
		$\beta_{\text{year},P}$	0.005	-0.002 – 0.011
	SD random intercepts	$\sigma_{b_{\text{int}}}$	0.933	0.798 – 1.149
	SD random slopes	$\sigma_{b_{\text{year}}}$	0.008	0.006 – 0.012
	SD residual	σ_{ε}	0.650	0.631 – 0.670
FN	Intercept	$\beta_{\text{int},B}$	8.991	8.479 – 9.457
		$\beta_{\text{int},G}$	7.616	7.136 – 8.030
		$\beta_{\text{int},P}$	5.478	4.928 – 6.027
	Year *(in fledglings per year)*	$\beta_{\text{year},B}$	-0.019	-0.028 – -0.011
		$\beta_{\text{year},G}$	-0.018	-0.025 – -0.010
		$\beta_{\text{year},P}$	0.000	-0.009 – 0.009
	SD random intercepts	$\sigma_{b_{\text{int}}}$	0.984	0.799 – 1.194
	SD random slopes	$\sigma_{b_{\text{year}}}$	0.013	0.010 – 0.018
	SD residual	σ_{ε}	0.977	0.949 – 1.004

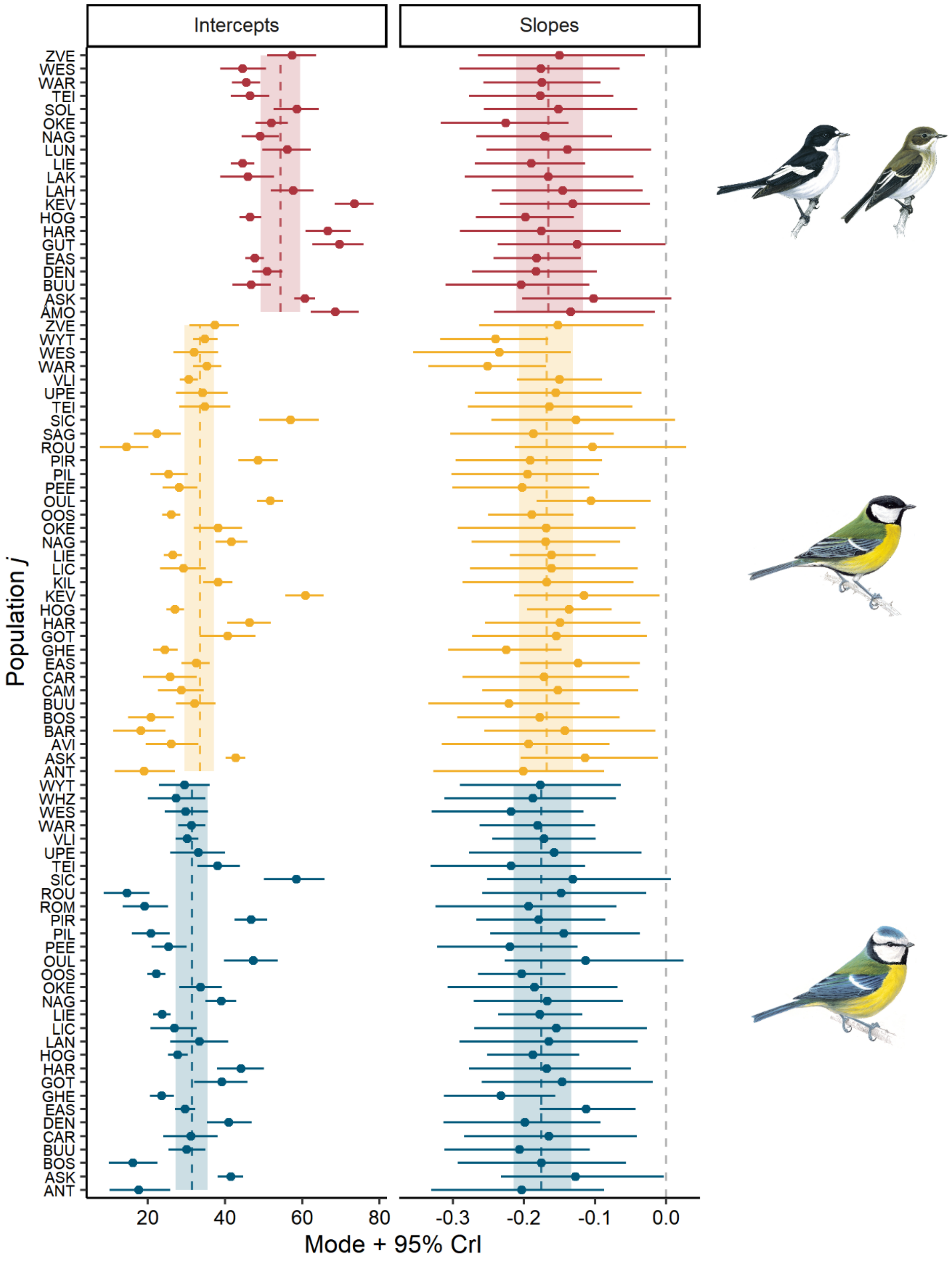

Figure S2. Population-specific intercepts ($\beta_{\text{int},j} + b_{\text{int},jk}$) and slopes ($\beta_{\text{year},j} + b_{\text{year},jk}$) from linear mixed-effects models of time trends in annual median laying date for blue tits (blue), great tits (yellow), and pied flycatchers (red). The analysis was based on 2,601 observations (years) from 85 populations. Estimates are given by the posterior mean and 95% confidence interval (95% CrI). Dashed lines and ribbons are the modes and 95% CrI for the intercept $\beta_{\text{int},j}$ and slope $\beta_{\text{year},j}$ per species j. Bird drawings reproduced with permission of Mike Langman, RSPB (rspb-images.com).

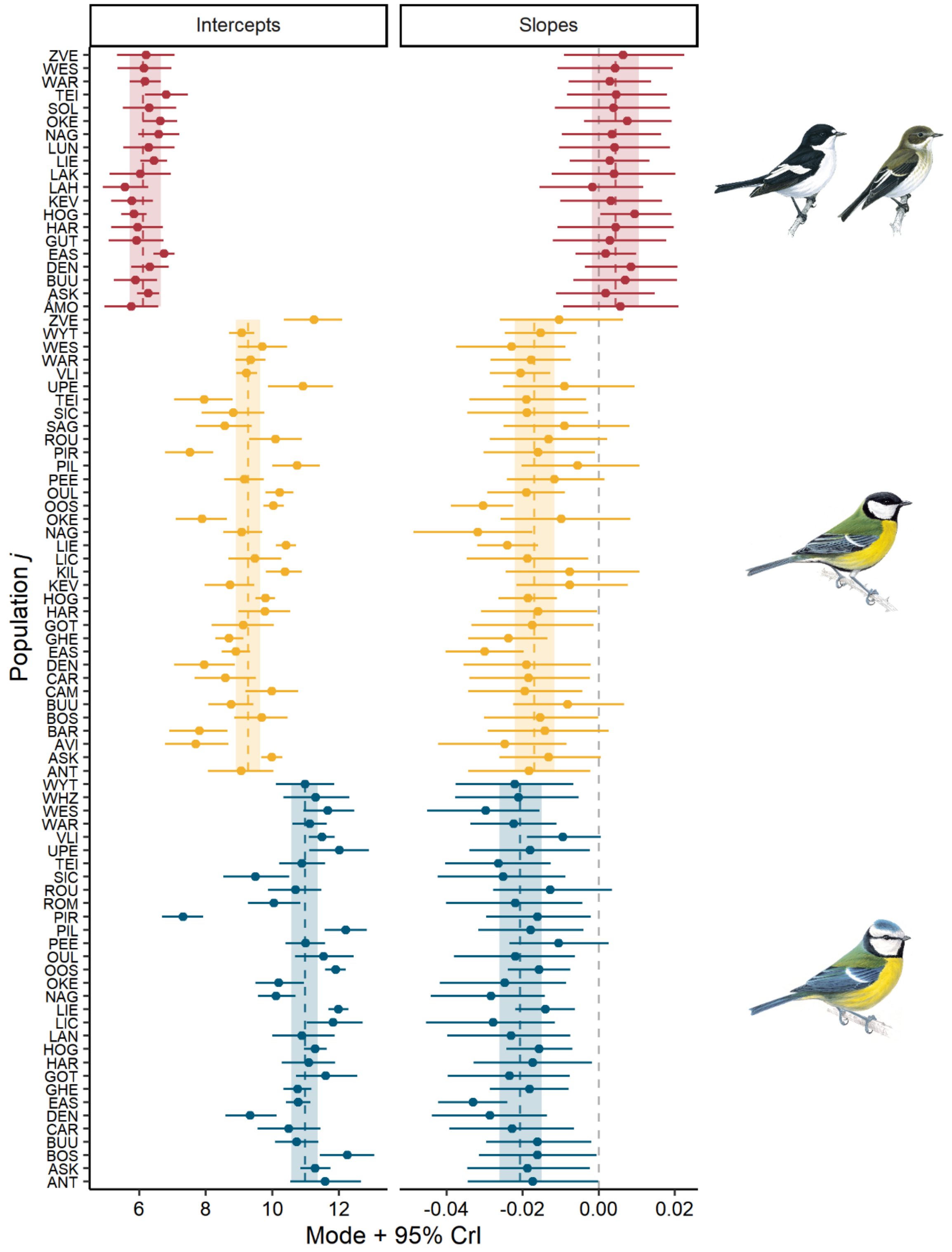

Figure S3. Population-specific intercepts ($\beta_{\text{int},j} + b_{\text{int},jk}$) and slopes ($\beta_{\text{year},j} + b_{\text{year},jk}$) from linear mixed-effects models of time trends in annual mean clutch size for blue tits (blue), great tits (yellow), and pied flycatchers (red). The analysis was based on 2,615 observations (years) from 86 populations. Estimates are given by the posterior mean and 95% confidence interval (95% CrI). Dashed lines and ribbons are the modes and 95% CrI for the intercept $\beta_{\text{int},j}$ and slope $\beta_{\text{year},j}$ per species k. Bird drawings reproduced with permission of Mike Langman, RSPB (rspb-images.com).

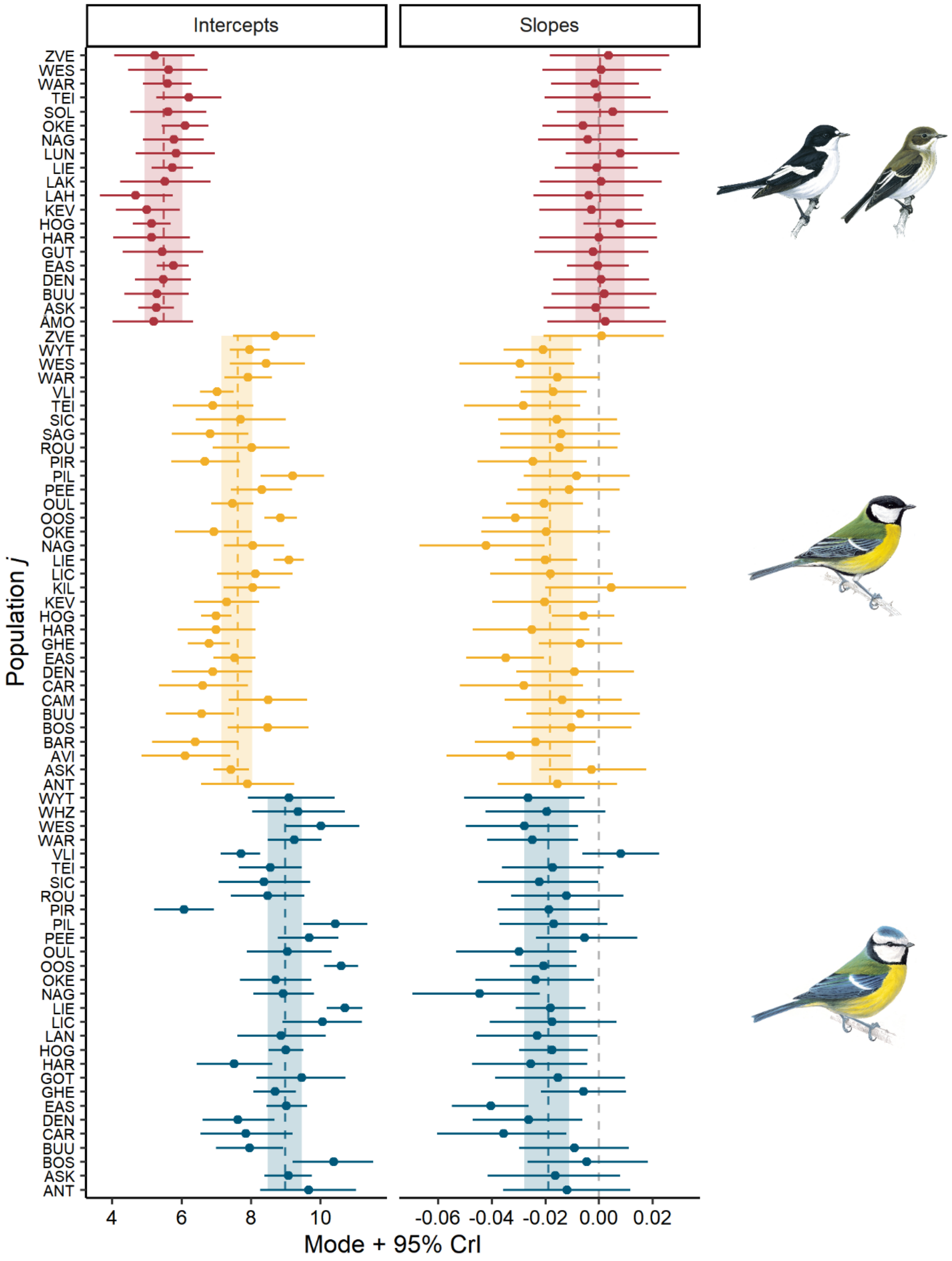

Figure S4. Population-specific intercepts ($\beta_{\text{int},j} + b_{\text{int},jk}$) and slopes ($\beta_{\text{year},j} + b_{\text{year},jk}$) from linear mixed-effects models of time trends in annual mean fledgling number for blue tits (blue), great tits (yellow), and pied flycatchers (red). The analysis was based on 2,522 observations (years) from 82 populations. Estimates are given by the posterior mode and 95% credible interval (95% CrI). Dashed lines and ribbons are the modes and 95% CrI for the intercept $\beta_{\text{int},j}$ and slope $\beta_{\text{year},j}$ per species j. Bird drawings reproduced with permission of Mike Langman, RSPB (rspb-images.com).

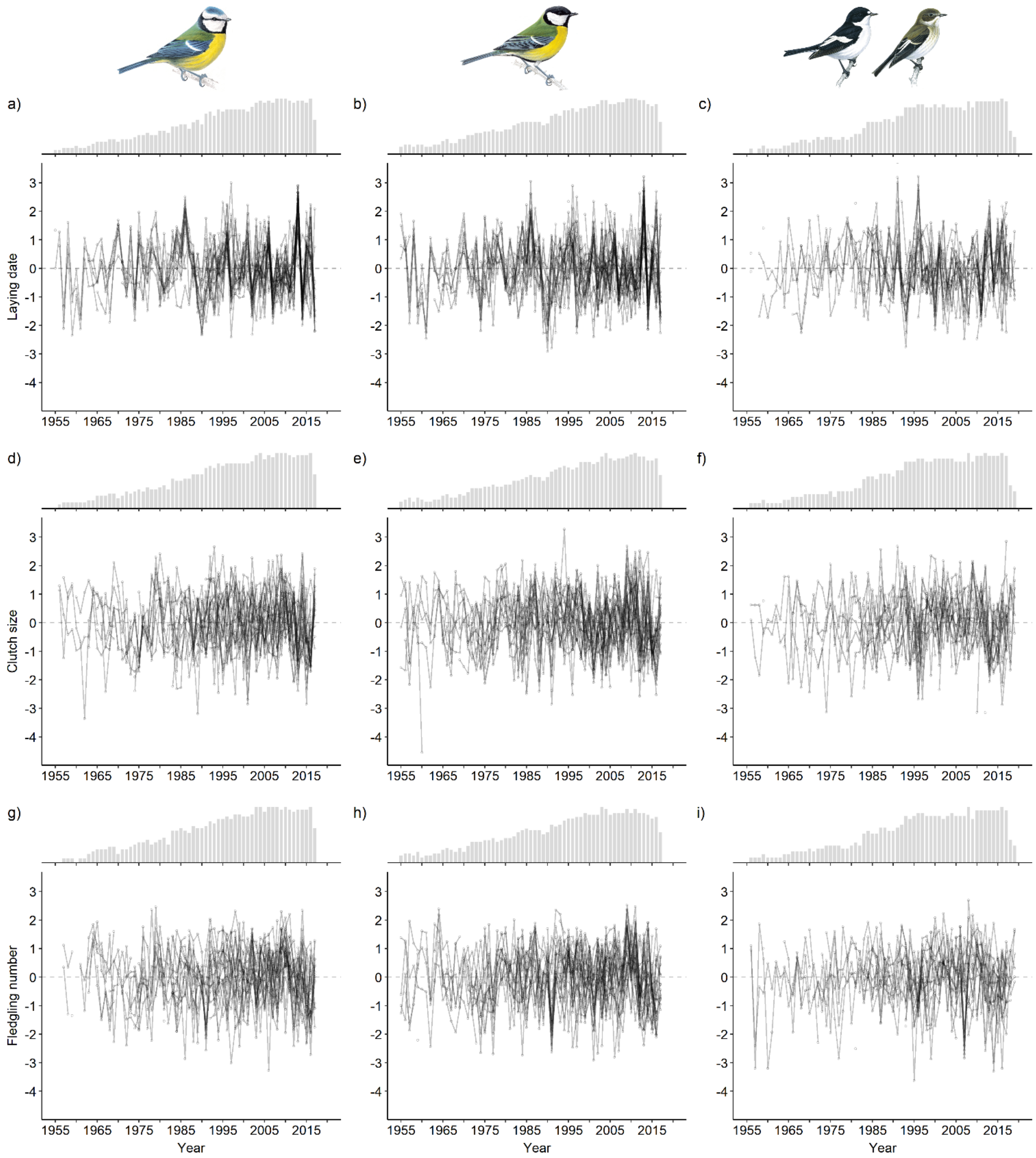

Figure S5. Temporal variation in laying date (a-c), clutch size (d-f), and fledgling number (g-i) of blue tit (a, d, g), great tit (b, e, h), and pied flycatcher (c, f, i) populations. Lines and points correspond to population detrended and normalized time series of annual average trait values (median laying dates, mean clutch sizes, and fledgling numbers), allowing years with missing data. Histograms show annual data density, i.e., the relative frequency of populations available per year. Bird drawings reproduced with permission of Mike Langman, RSPB (rspb-images.com).

Table S2. Outputs of linear mixed-effects models of mean temperature effects on average trait values of median laying date (LD), mean clutch size (CS), and mean fledgling number (FN) for blue tits (B), great tits (G), and pied flycatchers (P). Models included an intercept per species ($\beta_{\text{int},j}$), a slope for the linear time trend per species ($\beta_{\text{year},j}$), a slope for mean temperature per species ($\beta_{\text{temp},j}$), random intercepts per population ($b_{\text{int},jk}$), random slopes of the linear time trends per population ($b_{\text{year},jk}$), and random slopes for mean temperature per population ($b_{\text{temp},jk}$). The analyses were based on 2,601 observations from 85 populations for laying date, 2,615 observations from 86 populations for clutch size, and 2,522 observations from 82 populations for fledgling number. Estimates are given by the posterior mode and the 95% credible interval (95% CrI).

Trait	Parameter		Mode	95% CrI
LD	Intercept	$\beta_{\text{int},B}$	29.767	26.743 – 33.169
		$\beta_{\text{int},G}$	31.555	28.543 – 34.711
		$\beta_{\text{int},G}$	49.674	45.426 – 53.794
	Year *(in days per year)*	$\beta_{\text{year},B}$	-0.066	-0.105 – -0.017
		$\beta_{\text{year},G}$	-0.064	-0.100 – -0.021
		$\beta_{\text{year},P}$	-0.106	-0.152 – -0.059
	Temperature *(in days per SD of temperature (ºC))*	$\beta_{\text{temp},B}$	-11.718	-13.390 – -10.218
		$\beta_{\text{temp},G}$	-10.558	-11.977 – -8.921
		$\beta_{\text{temp},P}$	-5.614	-7.629 – -3.819
	SD random intercepts	$\sigma_{b_{\text{int}}}$	7.741	6.416 – 9.917
	SD random time slopes	$\sigma_{b_{\text{year}}}$	0.074	0.045 – 0.105
	SD random temperature slopes	$\sigma_{b_{\text{temp}}}$	3.391	2.627 – 4.488
	SD residual	σ_{ε}	4.070	3.956 – 4.189
CS	Intercept	$\beta_{\text{int},B}$	11.019	10.569 – 11.425
		$\beta_{\text{int},G}$	9.306	8.923 – 9.693
		$\beta_{\text{int},G}$	6.466	5.910 – 6.914
	Year *(in eggs per year)*	$\beta_{\text{year},B}$	-0.022	-0.028 – -0.016
		$\beta_{\text{year},G}$	-0.019	-0.024 – -0.013
		$\beta_{\text{year},P}$	0.003	-0.004 – 0.009
	Temperature *(in eggs per SD of temperature (ºC))*	$\beta_{\text{temp},B}$	0.158	-0.012 – 0.293
		$\beta_{\text{temp},G}$	0.155	0.028 – 0.287
		$\beta_{\text{temp},P}$	0.225	0.096 – 0.406
	SD random intercepts	$\sigma_{b_{\text{int}}}$	0.995	0.836 – 1.200
	SD random time slopes	$\sigma_{b_{\text{year}}}$	0.009	0.006 – 0.013
	SD random temperature slopes	$\sigma_{b_{\text{temp}}}$	0.021	0.003 – 0.189
	SD residual	σ_{ε}	0.646	0.629 – 0.664
FN	Intercept	$\beta_{\text{int},B}$	9.030	8.472 – 9.478
		$\beta_{\text{int},G}$	7.684	7.248 – 8.125
		$\beta_{\text{int},G}$	5.477	4.895 – 6.030
	Year *(in fledglings per year)*	$\beta_{\text{year},B}$	-0.021	-0.030 – -0.013
		$\beta_{\text{year},G}$	-0.021	-0.028 – -0.013
		$\beta_{\text{year},P}$	0.000	-0.008 – 0.010
	Temperature *(in fledglings per SD of temperature (ºC))*	$\beta_{\text{temp},B}$	0.148	-0.093 – 0.423
		$\beta_{\text{temp},G}$	0.209	-0.014 – 0.449
		$\beta_{\text{temp},P}$	-0.062	-0.317 – 0.248
	SD random intercepts	$\sigma_{b_{\text{int}}}$	0.955	0.788 – 1.206
	SD random time slopes	$\sigma_{b_{\text{year}}}$	0.014	0.009 – 0.018
	SD random temperature slopes	$\sigma_{b_{\text{temp}}}$	0.349	0.136 – 0.536
	SD residual	σ_{ε}	0.967	0.942 – 0.997

Table S3. Outputs of linear mixed-effects models of mean precipitation effects on average trait values of median laying date (LD), mean clutch size (CS), and mean fledgling number (FN) for blue tits (B), great tits (G), and pied flycatchers (P). Models included an intercept per species ($\beta_{\mathrm{int},j}$), a slope for the linear time trend per species ($\beta_{\mathrm{year},j}$),a slope for mean precipitation per species ($\beta_{\mathrm{prec},j}$), random intercepts per population ($b_{\mathrm{int},jk}$), random slopes of the linear time trends per population ($b_{\mathrm{year},jk}$), and random slopes for mean precipitation per population ($b_{\mathrm{prec},jk}$). The analyses were based on 2,601 observations from 85 populations for laying date, 2,615 observations from 86 populations for clutch size, and 2,522 observations from 82 populations for fledgling number. Estimates are given by the posterior mode and the 95% credible interval (95% CrI).

Trait	Parameter		Mode	95% CrI
LD	Intercept	$\beta_{\mathrm{int},B}$	31.349	27.299 – 35.517
		$\beta_{\mathrm{int},G}$	33.732	29.426 – 37.645
		$\beta_{\mathrm{int},G}$	53.850	49.417 – 59.443
	Year *(in days per year)*	$\beta_{\mathrm{year},B}$	-0.174	-0.218 – -0.132
		$\beta_{\mathrm{year},G}$	-0.177	-0.209 – -0.133
		$\beta_{\mathrm{year},P}$	-0.172	-0.209 – -0.118
	Precipitation *(in days per SD of precipitation (mm))*	$\beta_{\mathrm{prec},B}$	0.749	0.149 – 1.182
		$\beta_{\mathrm{prec},G}$	0.822	0.330 – 1.328
		$\beta_{\mathrm{prec},P}$	0.071	-0.468 – 0.609
	SD random intercepts	$\sigma_{b_{\mathrm{int}}}$	10.494	9.173 – 12.783
	SD random time slopes	$\sigma_{b_{\mathrm{year}}}$	0.057	0.033 – 0.089
	SD random precipitation slopes	$\sigma_{b_{\mathrm{prec}}}$	0.060	0.009 – 0.567
	SD residual	σ_{ε}	5.028	4.895 – 5.171
CS	Intercept	$\beta_{\mathrm{int},B}$	10.993	10.562 – 11.376
		$\beta_{\mathrm{int},G}$	9.301	8.895 – 9.639
		$\beta_{\mathrm{int},G}$	6.242	5.724 – 6.667
	Year *(in eggs per year)*	$\beta_{\mathrm{year},B}$	-0.020	-0.026 – -0.015
		$\beta_{\mathrm{year},G}$	-0.017	-0.022 – 0.011
		$\beta_{\mathrm{year},P}$	0.004	-0.002 – 0.010
	Precipitation *(in eggs per SD of precipitation (mm))*	$\beta_{\mathrm{prec},B}$	-0.005	-0.066 – 0.068
		$\beta_{\mathrm{prec},G}$	-0.008	-0.079 – 0.048
		$\beta_{\mathrm{prec},P}$	-0.029	-0.100 – 0.042
	SD random intercepts	$\sigma_{b_{\mathrm{int}}}$	0.918	0.797 – 1.144
	SD random time slopes	$\sigma_{b_{\mathrm{year}}}$	0.009	0.006 – 0.012
	SD random precipitation slopes	$\sigma_{b_{\mathrm{prec}}}$	0.014	0.002 – 0.092
	SD residual	σ_{ε}	0.650	0.633 – 0.669
FN	Intercept	$\beta_{\mathrm{int},B}$	8.949	8.485 – 9.435
		$\beta_{\mathrm{int},G}$	7.569	7.131 – 7.998
		$\beta_{\mathrm{int},G}$	5.444	4.937 – 6.020
	Year *(in fledglings per year)*	$\beta_{\mathrm{year},B}$	-0.019	-0.029 – -0.011
		$\beta_{\mathrm{year},G}$	-0.017	-0.025 – -0.009
		$\beta_{\mathrm{year},P}$	0.000	-0.009 – 0.010
	Precipitation *(in fledglings per SD of precipitation (mm))*	$\beta_{\mathrm{prec},B}$	0.026	-0.077 – 0.126
		$\beta_{\mathrm{prec},G}$	-0.048	-0.149 – 0.040
		$\beta_{\mathrm{prec},P}$	-0.045	-0.149 – 0.058
	SD random intercepts	$\sigma_{b_{\mathrm{int}}}$	0.982	0.798 – 1.206
	SD random time slopes	$\sigma_{b_{\mathrm{year}}}$	0.013	0.009 – 0.019
	SD random precipitation slopes	$\sigma_{b_{\mathrm{prec}}}$	0.014	0.001 – 0.120
	SD residual	σ_{ε}	0.977	0.949 – 1.005

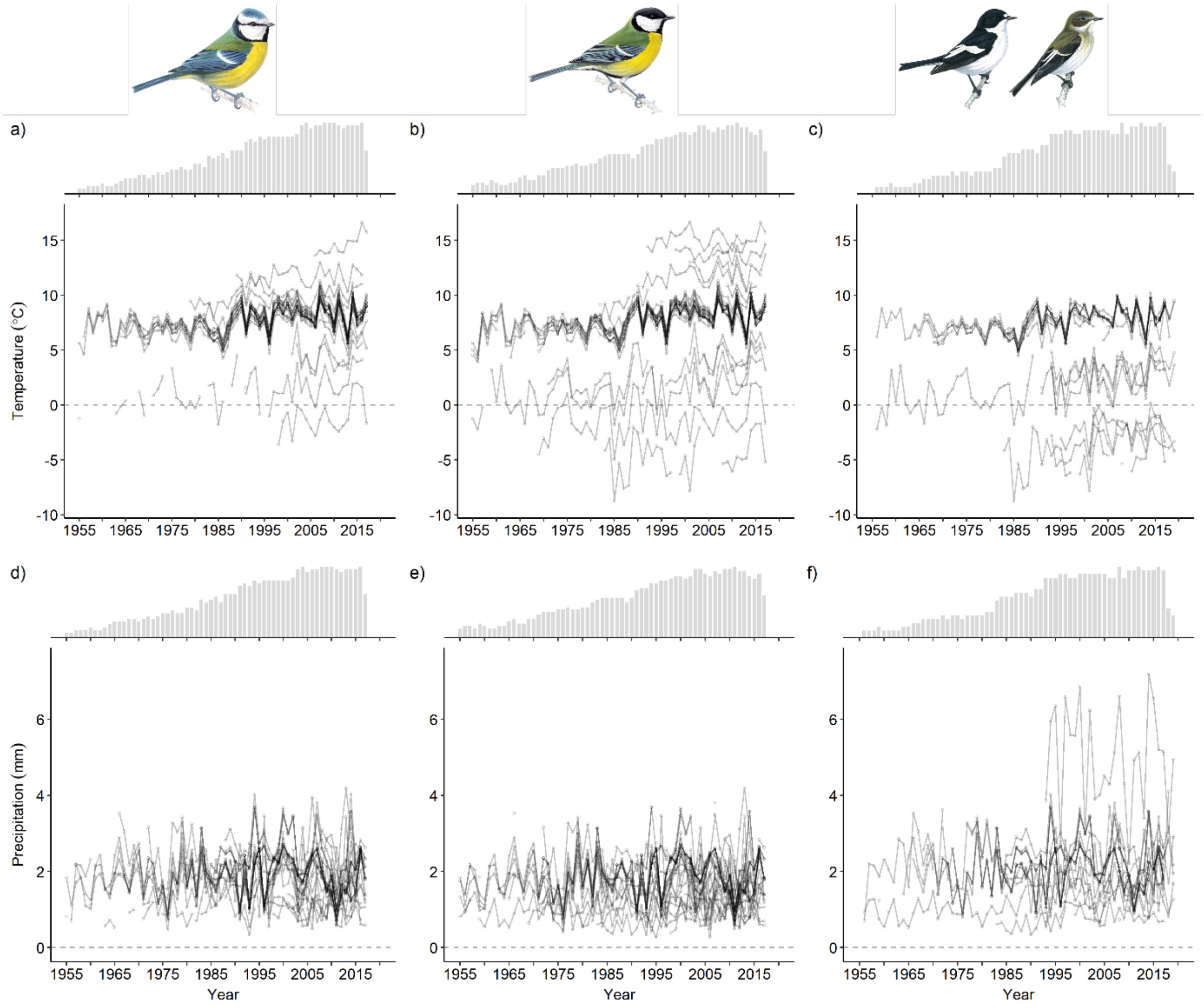

Figure S6. Temporal variation in mean temperature (a-c) and mean precipitation (d-f) across blue tit (a, d), great tit (b, e), and pied flycatcher (c, f) populations. Temperature and precipitation are local variables that are unique for each location. Lines and points correspond to annual means calculated from daily values in February-May, allowing years with missing data. Histograms show annual data density, i.e., the relative frequency of populations available per year. Bird drawings reproduced with permission of Mike Langman, RSPB (rspb-images.com).

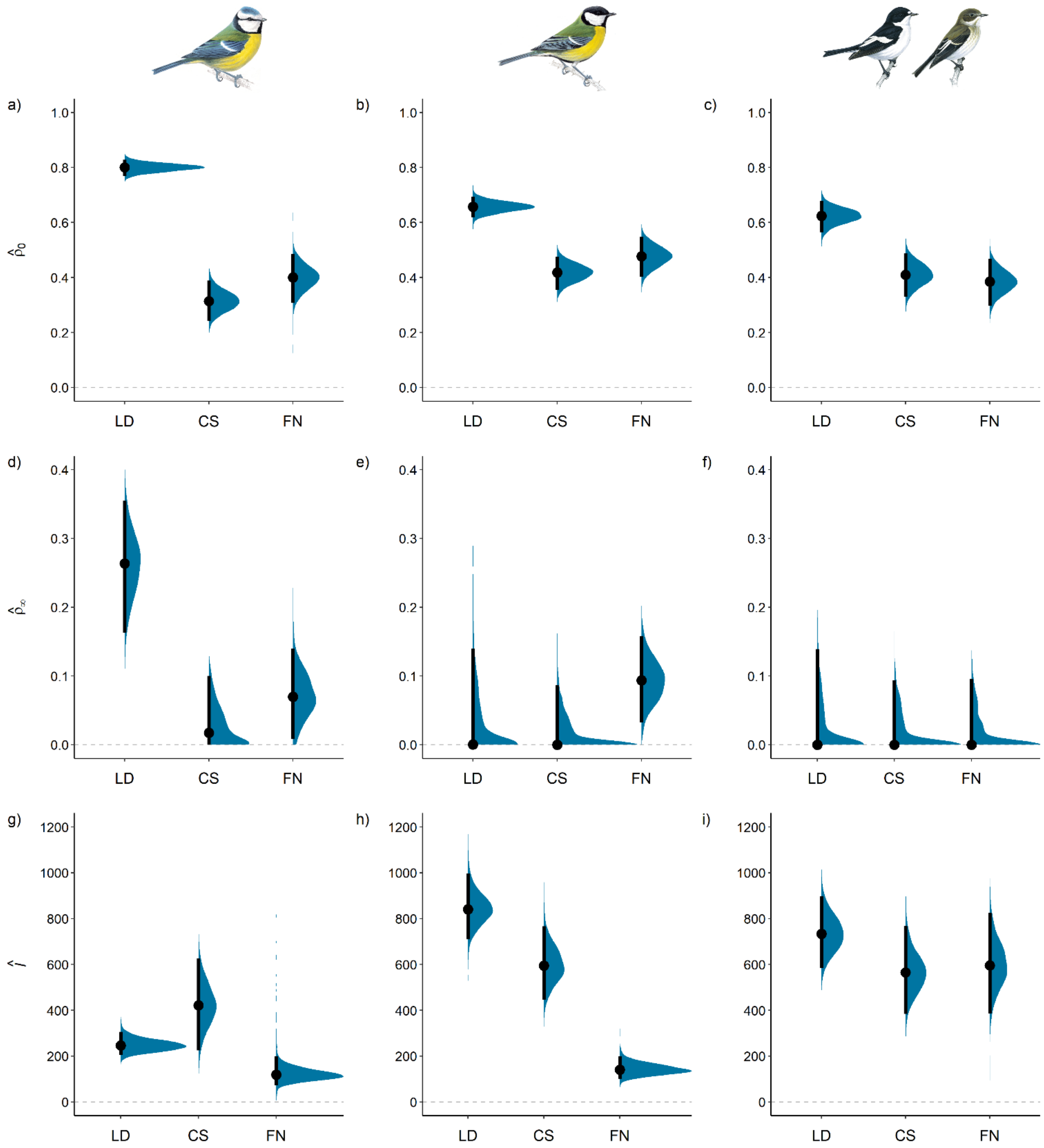

Figure S7. Bootstrap distributions of spatial synchrony parameter estimates, correlation at zero distance $\hat{\rho}_0$ (a-c), correlation at infinity $\hat{\rho}_\infty$ (d-f), and spatial scale $\hat{l}$ in km (g-i), for laying date (LD), clutch size (CS), and fledgling number (FN) in blue tit (a, d, g), great tit (b, e, h), and pied flycatcher (c, f, i) populations. Black dots are the median estimate, black bars the 95% confidence interval, and blue distributions the kernel density based on 2,000 bootstrap replicates. Spatial synchrony parameters were restricted to be positive. Bird drawings reproduced with permission of Mike Langman, RSPB (rspb-images.com).

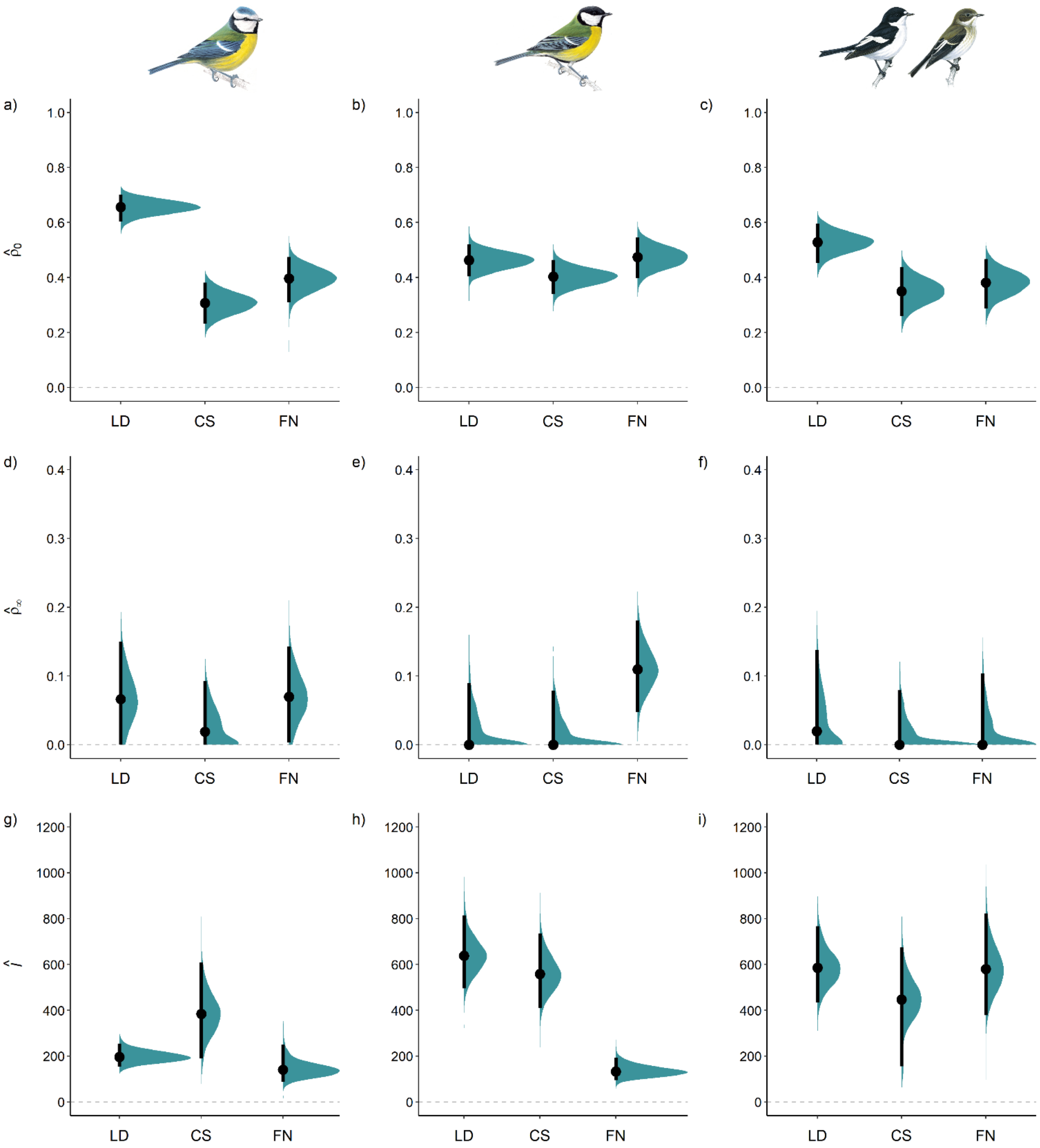

Figure S8. Bootstrap distributions of spatial synchrony parameter estimates, correlation at zero distance $\hat{\rho}_0$ (a-c), correlation at infinity $\hat{\rho}_\infty$ (d-f), and spatial scale $\hat{l}$ in km (g-i), for laying date (LD), clutch size (CS), and fledgling number (FN) in blue tit (a, d, g), great tit (b, e, h), and pied flycatcher (c, f, i) populations. Spatial synchrony was calculated on the residuals after accounting for the effects of mean temperature in February-May. Black dots are the median estimate, black bars the 95% confidence interval, and the teal distribution the kernel density based on 2,000 bootstrap replicates. Spatial synchrony parameters were restricted to be positive. Bird drawings reproduced with permission of Mike Langman, RSPB (rspb-images.com).

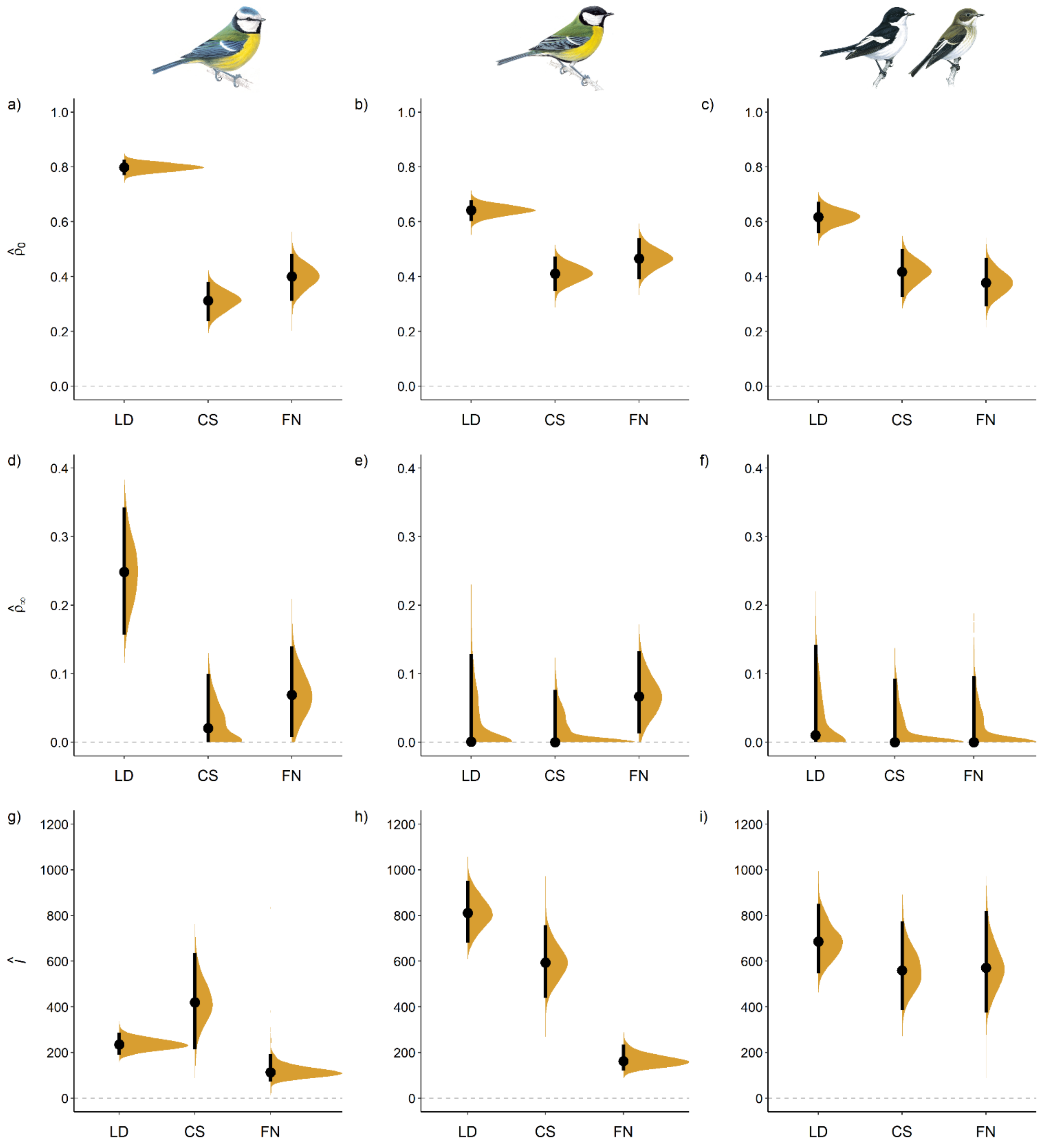

Figure S9. Bootstrap distributions of spatial synchrony parameter estimates, correlation at zero distance $\hat{\rho}_0$ (a-c), correlation at infinity $\hat{\rho}_\infty$ (d-f), and spatial scale $\hat{l}$ in km (g-i), for laying date (LD), clutch size (CS), and fledgling number (FN) in blue tit (a, d, g), great tit (b, e, h), and pied flycatcher (c, f, i) populations. Spatial synchrony was calculated on the residuals after accounting for the effects of mean precipitation in February-May. Black dots are the median estimate, black bars the 95% confidence interval, and the yellow distribution the kernel density based on 2,000 bootstrap replicates. Spatial synchrony parameters were restricted to be positive. Bird drawings reproduced with permission of Mike Langman, RSPB (rspb-images.com).
