# Supplementary material for: Temperature synchronizes temporal variation in laying dates across European hole‐nesting passerines
Source: Ecology. 2022 Dec 21;104(2):e3908. doi: 10.1002/ecy.3908 (PMC10078612; doi:10.1002/ecy.3908)
Supplement: Supplementary file 2 — Appendix S2 [file ECY-104-0-s001.pdf]

Supplementary Materials to

Temperature synchronizes temporal variation in laying dates across European hole-nesting passerines

Ecology

Stefan J.G. Vriend, Vidar Grøtan, Marlène Gamelon, Frank Adriaensen, Markus P. Ahola, Elena Álvarez, Liam D. Bailey, Emilio Barba, Jean-Charles Bouvier, Malcolm D. Burgess, Andrey Bushuev, Carlos Camacho, David Canal, Anne Charmantier, Ella F. Cole, Camillo Cusimano, Blandine F. Doligez, Szymon M. Drobniak, Anna Dubiec, Marcel Eens, Tapio Eeva, Kjell Einar Erikstad, Peter N. Ferns, Anne E. Goodenough, Ian R. Hartley, Shelley A. Hinsley, Elena Ivankina, Rimvydas Juškaitis, Bart Kempenaers, Anvar B. Kerimov, John Atle Kålås, Claire Lavigne, Agu Leivits, Mark C. Mainwaring, Jesús Martínez-Padilla, Erik Matthysen, Kees van Oers, Markku Orell, Rianne Pinxten, Tone Kristin Reiertsen, Seppo Rytkönen, Juan Carlos Senar, Ben C. Sheldon, Alberto Sorace, János Török, Emma Vatka, Marcel E. Visser, Bernt-Erik Sæther

Appendix S2: Effect of North Atlantic Oscillation index on temporal variation and spatial synchrony in fitness-related traits

In addition to the two local climatic variables used in the main text, we used the North Atlantic Oscillation (NAO) index as a climatic variable on a larger, regional scale. We extracted daily data on the NAO-index from the Climate Prediction Center of the National Weather Service (www.cpc.ncep.noaa.gov) as follows:

1. Visit https://www.cpc.ncep.noaa.gov/products/precip/CWlink/pna/nao.shtml
2. Click on "Daily NAO index since January 1950"
3. Download "norm.daily.nao.cdas.z500.19500101_current.csv"

The NAO is a weather phenomenon expressed by an index based on the difference in surface sea-level pressures between the subtropical high-pressure center over the Azores and the subpolar low-pressure center over Iceland and drives the interannual fluctuations in temperature and precipitation (Hurrell 1995). Positive NAO phases are associated with warmer, wetter weather and advanced springs in northern Europe and warmer, drier weather and advanced springs in southern Europe (Post & Stenseth 1999; Gordo & Sanz 2010). In contrast to the local climatic variables used in this study, the NAO index is the same for all populations per year. Temporal variation in the NAO index is shown in Figure S1.

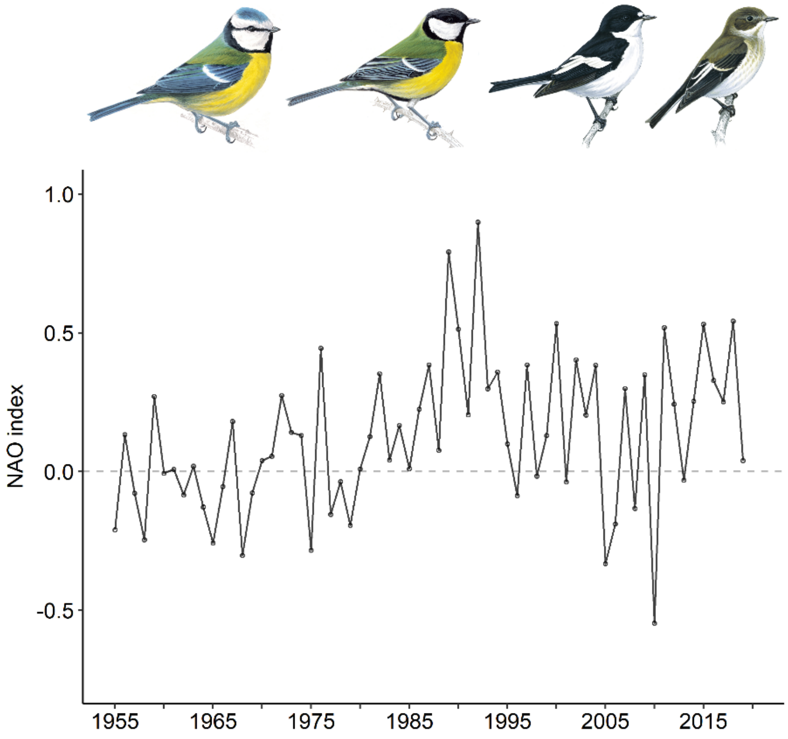

Figure S1. Temporal variation in the North Atlantic Oscillation (NAO) index. The NAO index is a regional variable that is the same for each species and population in contrast to temperature and precipitation (see Appendix S1: Figure S6). Lines and dots correspond to annual means calculated from daily values in February-May. Bird drawings reproduced with permission of Mike Langman, RSPB (rspb-images.com).

Effects of the NAO index on temporal variation in fitness-related trait values

The NAO index was included in two analyses. First, we explored the effects of the NAO index on the average trait values (i.e., median laying date, mean clutch size, and mean fledgling number) using eq. 2 in the main text. Annual values of the NAO index, calculated as the mean in February-May, were normalized (i.e., subtracting the mean and dividing by the standard deviation).

The effects of the NAO index were towards earlier laying for all three species, but stronger for blue tits and great tits than for pied flycatchers ($\beta_{\mathrm{NAO},j}$, Table S1). We found no evidence for an effect of the NAO index on clutch sizes and fledgling numbers in any species, except for a weak effect towards larger clutches for great tits ($\beta_{\mathrm{NAO},G}$, Table S1). In general, the effects of the NAO index were less strong and less variable among populations compared to the effects of mean temperature in February-May (Appendix S1: Table S2) and similar to the effects of mean precipitation in February-May (Appendix S1: Table S3).

Effects of the NAO index on spatial synchrony in fitness-related trait values

Second, we examined the extent to which the NAO index contributed to spatial synchrony in average trait values. We regressed the population-specific annual average trait values against the annual means of the NAO index using separate linear regression models for each combination of species and trait. The residuals were normalized and used in the spatial autocorrelation model (eq. 3 in the main text; 3 species, 3 traits, 9 models in total) to calculate the spatial synchrony in trait values after accounting for the effect of the NAO index.

Accounting for the NAO index in February-May had no effect on the spatial synchrony in trait values in any species-trait combination (Figure S2). The spatial synchrony parameter estimates from a model after accounting for the effects of the NAO index in February-May were similar (Figure S3) to the parameter estimates from the model without accounting for the effects of climatic variables (Appendix S1: Figure S7).

Table S1. Outputs of linear mixed-effects models of NAO index effects on average trait values of median laying date (LD), mean clutch size (CS), and mean fledgling number (FN) for blue tits (B), great tits (G), and pied flycatchers (P). Models included an intercept per species ($\beta_{\mathrm{int},j}$), a slope for the linear time trend per species ($\beta_{\mathrm{year},j}$),a slope for the NAO index per species ($\beta_{\mathrm{NAO},j}$), random intercepts per population ($b_{\mathrm{int},jk}$), random slopes of the linear time trends per population ($b_{\mathrm{year},jk}$), and random slopes for the NAO index per population ($b_{\mathrm{NAO},jk}$). The analyses were based on 2,601 observations from 85 populations for laying date, 2,615 observations from 86 populations for clutch size, and 2,522 observations from 82 populations for fledgling number. Estimates are given by the posterior mode and the 95% credible interval (95% CrI).

Trait	Parameter		Mode	95% CrI
LD	Intercept	$\beta_{\mathrm{int},B}$	30.949	27.113 – 35.261
		$\beta_{\mathrm{int},G}$	33.240	29.035 – 36.678
		$\beta_{\mathrm{int},G}$	53.855	49.312 – 59.305
	Year *(in days per year)*	$\beta_{\mathrm{year},B}$	-0.170	-0.210 – -0.120
		$\beta_{\mathrm{year},G}$	-0.161	-0.197 – -0.118
		$\beta_{\mathrm{year},P}$	-0.167	-0.209 – -0.108
	NAO index *(in days per SD of NAO-index)*	$\beta_{\mathrm{NAO},B}$	-1.241	-1.537 – -0.877
		$\beta_{\mathrm{NAO},G}$	-1.071	-1.410 – -0.766
		$\beta_{\mathrm{NAO},P}$	-0.473	-0.860 – -0.071
	SD random intercepts	$\sigma_{b_{\mathrm{int}}}$	10.515	8.965 – 12.571
	SD random time slopes	$\sigma_{b_{\mathrm{year}}}$	0.068	0.041 – 0.099
	SD random NAO index slopes	$\sigma_{b_{\mathrm{NAO}}}$	0.046	0.006 – 0.417
	SD residual	σ_{ε}	4.922	4.804 – 5.080
CS	Intercept	$\beta_{\mathrm{int},B}$	10.977	10.561 – 11.367
		$\beta_{\mathrm{int},G}$	9.281	8.895 – 9.660
		$\beta_{\mathrm{int},G}$	6.134	5.707 – 6.645
	Year *(in eggs per year)*	$\beta_{\mathrm{year},B}$	-0.020	-0.026 – -0.015
		$\beta_{\mathrm{year},G}$	-0.017	-0.023 – -0.012
		$\beta_{\mathrm{year},P}$	0.004	-0.002 – 0.011
	NAO index *(in eggs per SD of NAO-index)*	$\beta_{\mathrm{NAO},B}$	-0.018	-0.057 – 0.034
		$\beta_{\mathrm{NAO},G}$	0.045	0.010 – 0.092
		$\beta_{\mathrm{NAO},P}$	0.029	-0.021 – 0.079
	SD random intercepts	$\sigma_{b_{\mathrm{int}}}$	0.929	0.788 – 1.148
	SD random time slopes	$\sigma_{b_{\mathrm{year}}}$	0.009	0.006 – 0.013
	SD random NAO index slopes	$\sigma_{b_{\mathrm{NAO}}}$	0.006	0.001 – 0.059
	SD residual	σ_{ε}	0.649	0.631 – 0.668
FN	Intercept	$\beta_{\mathrm{int},B}$	8.945	8.475 – 9.424
		$\beta_{\mathrm{int},G}$	7.587	7.175 – 8.042
		$\beta_{\mathrm{int},G}$	5.500	4.920 – 6.009
	Year *(in fledglings per year)*	$\beta_{\mathrm{year},B}$	-0.019	-0.028 – -0.011
		$\beta_{\mathrm{year},G}$	-0.018	-0.026 – -0.010
		$\beta_{\mathrm{year},P}$	0.000	-0.009 – 0.009
	NAO index *(in fledglings per SD of NAO-index)*	$\beta_{\mathrm{NAO},B}$	-0.056	-0.127 – 0.010
		$\beta_{\mathrm{NAO},G}$	0.043	-0.025 – 0.103
		$\beta_{\mathrm{NAO},P}$	0.010	-0.068 – 0.092
	SD random intercepts	$\sigma_{b_{\mathrm{int}}}$	0.979	0.784 – 1.194
	SD random time slopes	$\sigma_{b_{\mathrm{year}}}$	0.013	0.009 – 0.018
	SD random NAO index slopes	$\sigma_{b_{\mathrm{NAO}}}$	0.012	0.002 – 0.100
	SD residual	σ_{ε}	0.974	0.948 – 1.004

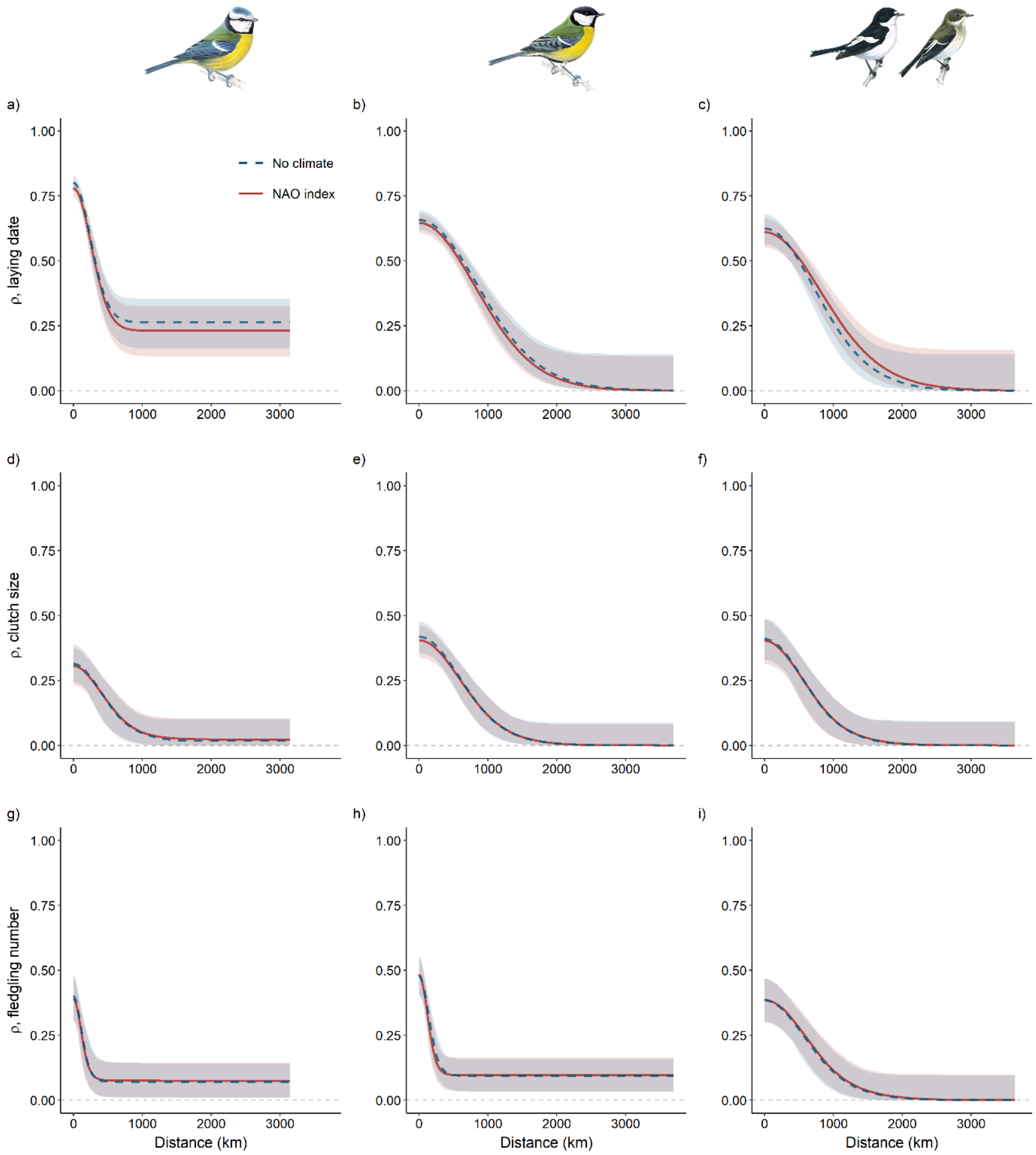

Figure S2. Spatial synchrony in laying date (a-c), clutch size (d-f), and fledgling number (g-i) of blue tit (a, d, g), great tit (b, e, h), and pied flycatcher (c, f, i) populations in relation to the distance (in km) between populations. Blue, dashed lines are the spatial synchrony in the traits without accounting for the effects of climatic variables (see Figure 3), red lines are the spatial synchrony in the residuals after accounting for the effects of the NAO index in February-May. Lines are the median and ribbons the 95% confidence interval based on 2,000 bootstrap replicates. Spatial synchrony parameters ($\hat{\rho}_0$, $\hat{\rho}_\infty$, and $\hat{l}$) were restricted to be positive. Bird drawings reproduced with permission of Mike Langman, RSPB (rspb-images.com).

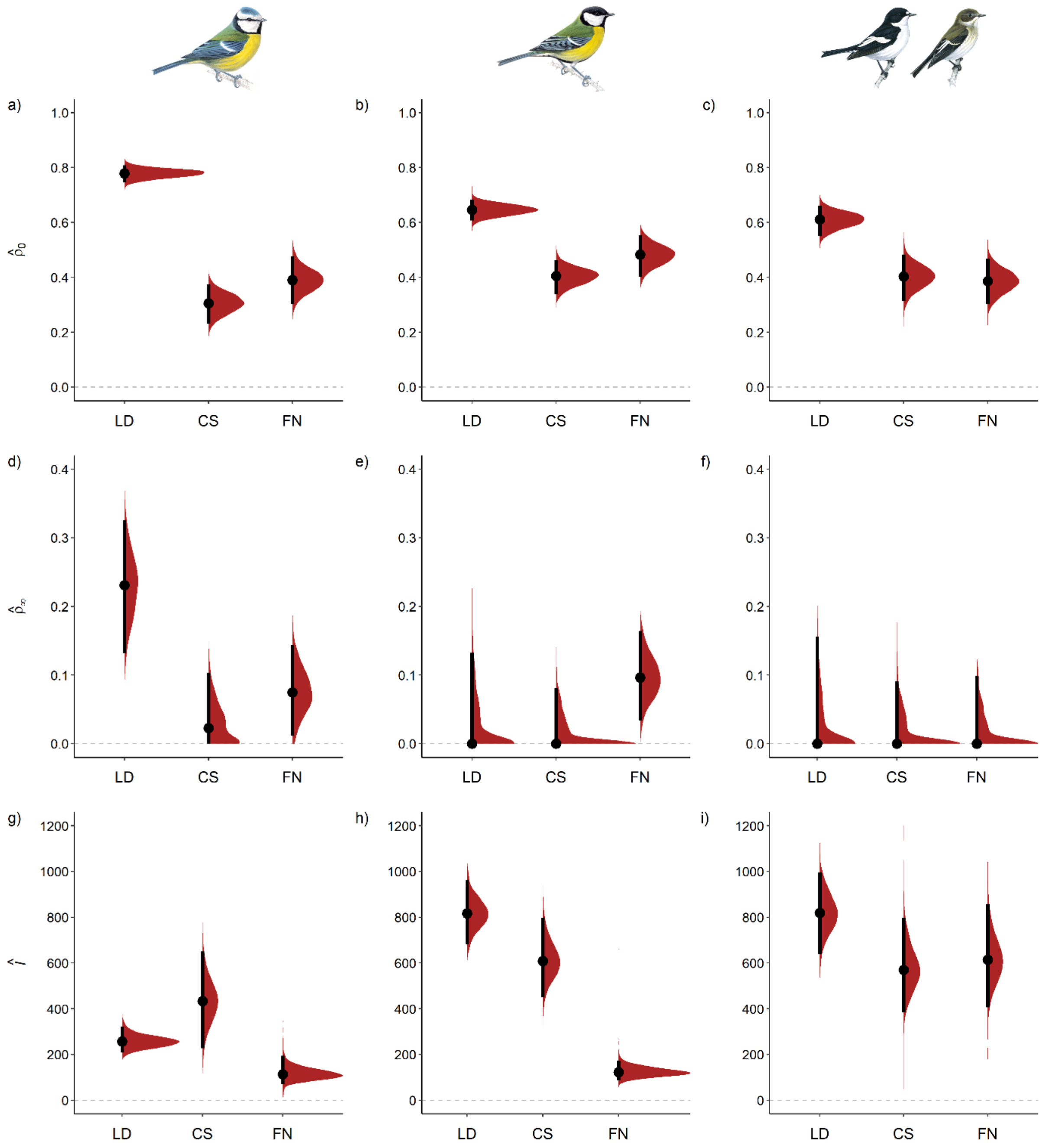

Figure S3. Bootstrap distributions of spatial synchrony parameter estimates, correlation at zero distance $\hat{\rho}_0$ (a-c), correlation at infinity $\hat{\rho}_\infty$ (d-f), and spatial scale $\hat{l}$ in km (g-i), for laying date (LD), clutch size (CS), and fledgling number (FN) in blue tit (a, d, g), great tit (b, e, h), and pied flycatcher (c, f, i) populations. Spatial synchrony was calculated on the residuals after accounting for the effects of the NAO index in February-May. Black dots are the median estimate, black bars the 95% confidence interval, and blue distributions the kernel density based on 2,000 bootstrap replicates. Spatial synchrony parameters were restricted to be positive. Bird drawings reproduced with permission of Mike Langman, RSPB (rspb-images.com).

Literature Cited

Gordo, O. & Sanz, J.J. (2010). Impact of climate change on plant phenology in Mediterranean ecosystems. *Glob. Chang. Biol.*, 16, 1082–1106.

Hurrell, J.W. (1995). Decadal trends in the North Atlantic oscillation: regional temperatures and precipitation. *Science*, 269, 676–679.

Post, E. & Stenseth, N.C. (1999). Climatic variability, plant phenology, and northern ungulates. *Ecology*, 80, 1322–1339.
