# Supplementary material for: Temperature synchronizes temporal variation in laying dates across European hole‐nesting passerines
Source: Ecology. 2022 Dec 21;104(2):e3908. doi: 10.1002/ecy.3908 (PMC10078612; doi:10.1002/ecy.3908)
Supplement: Supplementary file 3 — Appendix S3 [file ECY-104-0-s003.pdf]

Supplementary Materials to

Temperature synchronizes temporal variation in laying dates across European hole-nesting passerines

Ecology

Stefan J.G. Vriend, Vidar Grøtan, Marlène Gamelon, Frank Adriaensen, Markus P. Ahola, Elena Álvarez, Liam D. Bailey, Emilio Barba, Jean-Charles Bouvier, Malcolm D. Burgess, Andrey Bushuev, Carlos Camacho, David Canal, Anne Charmantier, Ella F. Cole, Camillo Cusimano, Blandine F. Doligez, Szymon M. Drobniak, Anna Dubiec, Marcel Eens, Tapio Eeva, Kjell Einar Erikstad, Peter N. Ferns, Anne E. Goodenough, Ian R. Hartley, Shelley A. Hinsley, Elena Ivankina, Rimvydas Juškaitis, Bart Kempenaers, Anvar B. Kerimov, John Atle Kålås, Claire Lavigne, Agu Leivits, Mark C. Mainwaring, Jesús Martínez-Padilla, Erik Matthysen, Kees van Oers, Markku Orell, Rianne Pinxten, Tone Kristin Reiertsen, Seppo Rytkönen, Juan Carlos Senar, Ben C. Sheldon, Alberto Sorace, János Török, Emma Vatka, Marcel E. Visser, Bernt-Erik Sæther

Appendix S3: Spatial synchrony in fledging success

As fledgling number is constrained by the initial size of the clutch, the temporal variation in clutch size and fledgling number might be non-independent. By considering fledgling number proportional to the size of the clutch (i.e., fledgling success or proportion fledged, calculated as fledgling number / clutch size), its spatiotemporal variation is more independent from that of clutch size.

Following the procedure described in section *Effects of climatic variables on spatial synchrony in fitness-related trait values* in the main text, we linearly detrended (i.e., retaining residuals from a linear regression of average trait value against year) and normalized the annual average values of fledgling success for each population. These detrended and normalized values were used in the spatial autocorrelation model (eq. 3 in the main text; 3 species, 3 models in total) to calculate the spatial synchrony in fledgling success.

Comparing the results of fledgling success to those of fledgling number, we see that their spatial synchrony patterns are very similar for all three species (Figure S1). Generally, spatial synchrony in the fledgling success seemed lower than in fledgling number, but 95% confidence intervals overlap, and the spatial scales l are of similar order of magnitude (blue tit: 119 km (fledgling number) vs. 39.1 km (fledgling success); great tit: 141 km vs. 187 km; pied flycatcher: 596 km vs. 563 km).

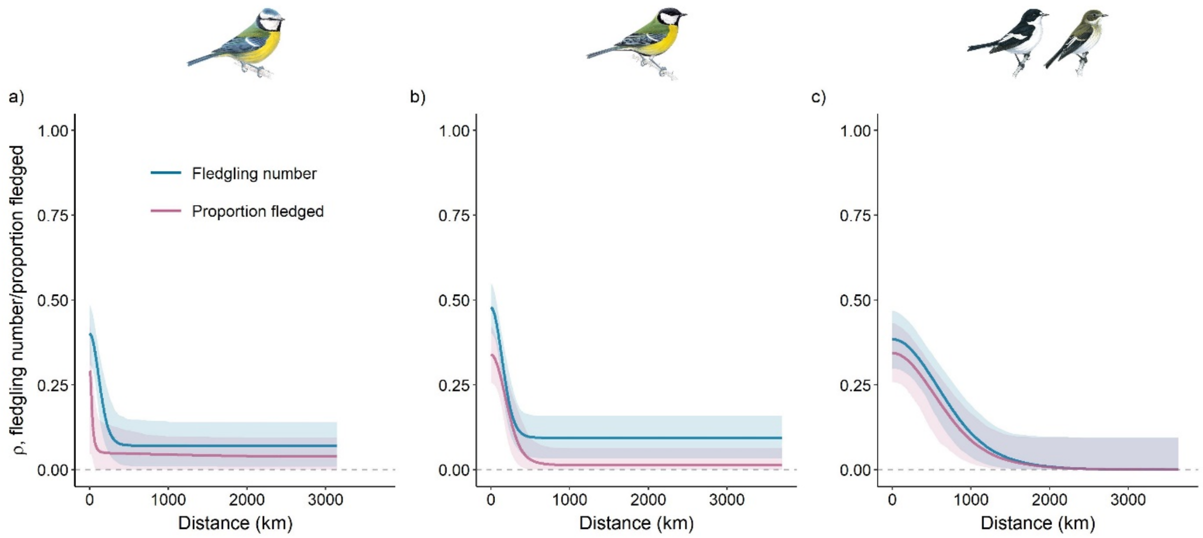

Figure S1. Spatial synchrony in fledgling number (blue) and fledgling success (i.e., fledgling number/clutch size; red) of blue tit (a), great tit (b), and pied flycatcher (c) populations in relation to distance (in km) between populations. Solid lines are the median and ribbons the 95% confidence interval based on 2,000 bootstrap replicates. Spatial synchrony parameters ($\hat{\rho}_0$, $\hat{\rho}_\infty$, and $\hat{l}$) were restricted to be positive. Bird drawings reproduced with permission of Mike Langman, RSPB (rspb-images.com).
