# Supplementary material for: Temperature synchronizes temporal variation in laying dates across European hole‐nesting passerines
Source: Ecology. 2022 Dec 21;104(2):e3908. doi: 10.1002/ecy.3908 (PMC10078612; doi:10.1002/ecy.3908)
Supplement: Supplementary file 4 — Appendix S4 [file ECY-104-0-s002.pdf]

Supplementary Materials to

Temperature synchronizes temporal variation in laying dates across European hole-nesting passerines

Ecology

Stefan J.G. Vriend, Vidar Grøtan, Marlène Gamelon, Frank Adriaensen, Markus P. Ahola, Elena Álvarez, Liam D. Bailey, Emilio Barba, Jean-Charles Bouvier, Malcolm D. Burgess, Andrey Bushuev, Carlos Camacho, David Canal, Anne Charmantier, Ella F. Cole, Camillo Cusimano, Blandine F. Doligez, Szymon M. Drobniak, Anna Dubiec, Marcel Eens, Tapio Eeva, Kjell Einar Erikstad, Peter N. Ferns, Anne E. Goodenough, Ian R. Hartley, Shelley A. Hinsley, Elena Ivankina, Rimvydas Juškaitis, Bart Kempenaers, Anvar B. Kerimov, John Atle Kålås, Claire Lavigne, Agu Leivits, Mark C. Mainwaring, Jesús Martínez-Padilla, Erik Matthysen, Kees van Oers, Markku Orell, Rianne Pinxten, Tone Kristin Reiertsen, Seppo Rytkönen, Juan Carlos Senar, Ben C. Sheldon, Alberto Sorace, János Török, Emma Vatka, Marcel E. Visser, Bernt-Erik Sæther

Appendix S4: Exploring the effect of southern populations

The studied populations of blue tits (*Cyanistes caeruleus*), great tits (*Parus major*), and pied flycatchers (*Ficedula hypoleuca*) represent a large part of each species' breeding range. Populations at the southern range margin generally experience the warmest average temperatures and may be more likely to encounter temperature extremes. Consequently, these populations may disproportionally influence the relationships between temperature and trait variation that we presented in the main text. To test this, we re-ran the temporal and climatic variation analysis (eq. 2 in the main text) and the spatial synchrony analysis (eq. 3 in the main text) on a data subset excluding southern populations (i.e., south of 45° N), and compared these results to the results of the analyses performed on the full dataset.

Comparing the results of the linear mixed-effects models of the full dataset to the results of the subset, we see that the estimates (mode + 95% credible intervals) for both the time trends in average trait values (β_{year}) and the effects of temperature on average trait values (β_{temp}) are very similar for all species-trait combinations (Figure S1).

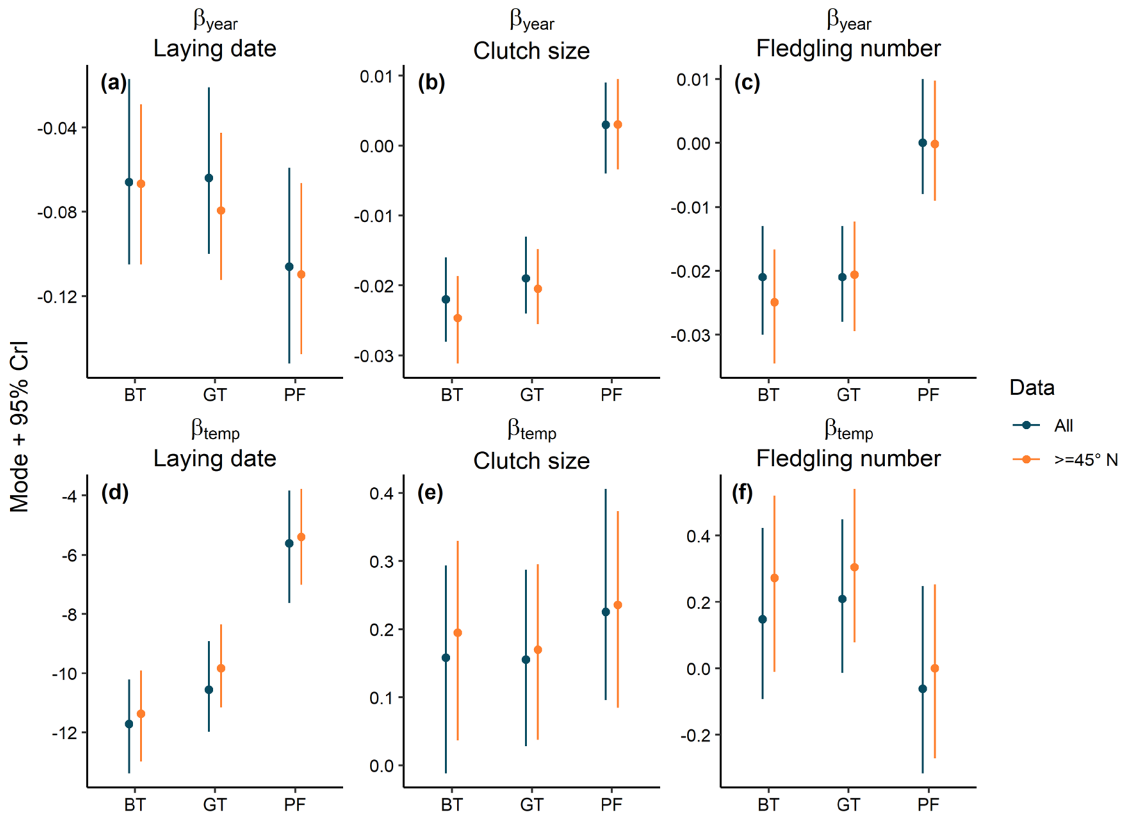

Figure S1. Estimates (posterior mode + 95% credible interval) of linear mixed-effects models of time trends (β_{year}) and mean temperature (β_{temp}) effects on values of laying date (a, d), clutch size (b, e), and fledgling number (c, f) for blue tits (BT), great tits (GT), and pied flycatchers (PF). Estimates for the full dataset are in blue and identical to the values in Appendix S1: Table S2; estimates for the dataset excluding southern populations are in orange.

Comparing the patterns of spatial synchrony in traits between the two datasets, both without (Figure S2) and after accounting for the effects of mean temperature (Figure S3), we again see that the results are very similar. Note that excluding southern populations reduced the maximum distance between pairs of populations in the subset; hence, the shorter

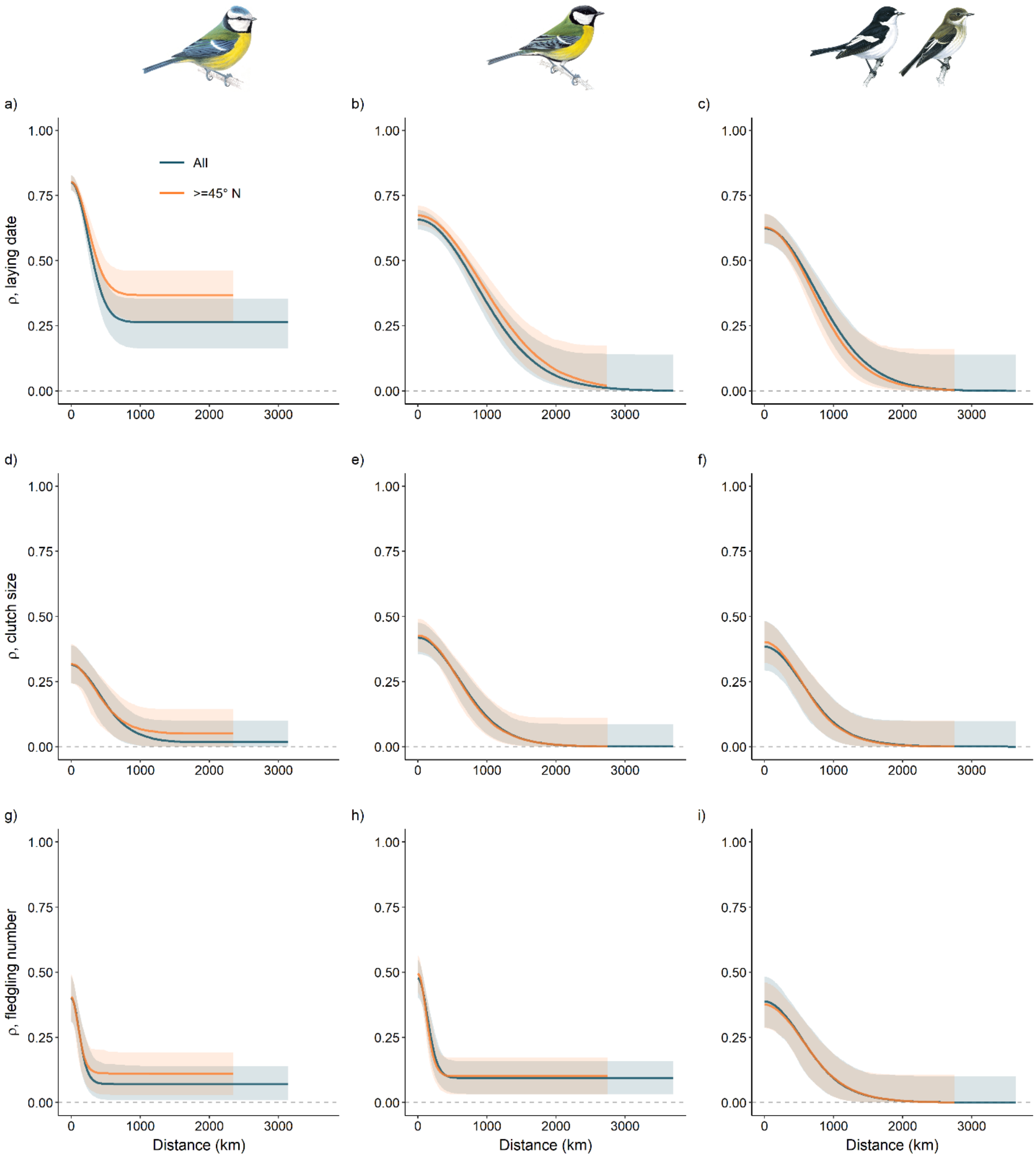

Figure S2. Spatial synchrony in laying date (a-c), clutch size (d-f), and fledgling number (g-i) of blue tit (a, d, g), great tit (b, e, h), and pied flycatcher (c, f, i) populations in relation to distance (in km). Spatial synchrony patterns for the full dataset are in blue and identical to the blue lines in Figure 3 of the main text; spatial synchrony patterns for the subset excluding southern populations are in orange. Bird drawings reproduced with permission of Mike Langman, RSPB (rspb-images.com).

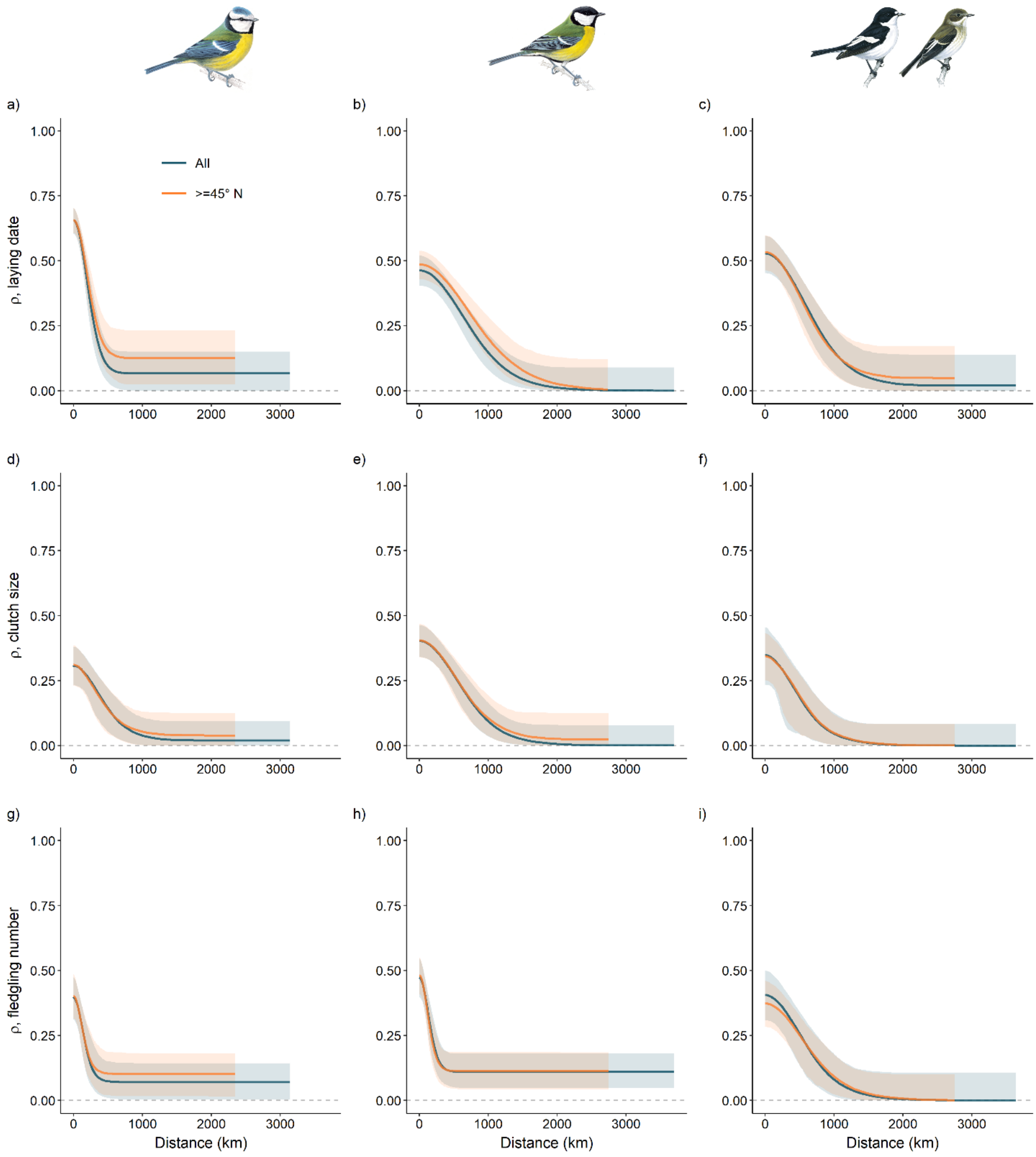

Figure S3. Spatial synchrony in laying date (a-c), clutch size (d-f), and fledgling number (g-i) of blue tit (a, d, g), great tit (b, e, h), and pied flycatcher (c, f, i) populations in relation to distance (in km) after accounting for the effects of mean temperature. Spatial synchrony patterns for the full dataset are in blue and identical to the teal lines in Figure 4 of the main text; spatial synchrony patterns for the subset excluding southern populations are in orange. Bird drawings reproduced with permission of Mike Langman, RSPB (rspb-images.com).

The comparison of spatial synchrony patterns between the two datasets showed a slight discrepancy in $\hat{\rho}_\infty$ for blue tit laying dates (Figure S2a). This discrepancy could not be attributed to different time trends in laying dates (β_{year}, Figure S1a), different effects of temperature on laying dates (β_{temp}, Figure S1d) or a different influence of mean temperature on the spatial synchrony in laying dates (Figure R3a) in southern populations, because these effects did not vary between the datasets. Exploring the cause of this discrepancy further, we found that this seemed to relate to the relatively low correlations between northern and southern populations instead (Figure S4).

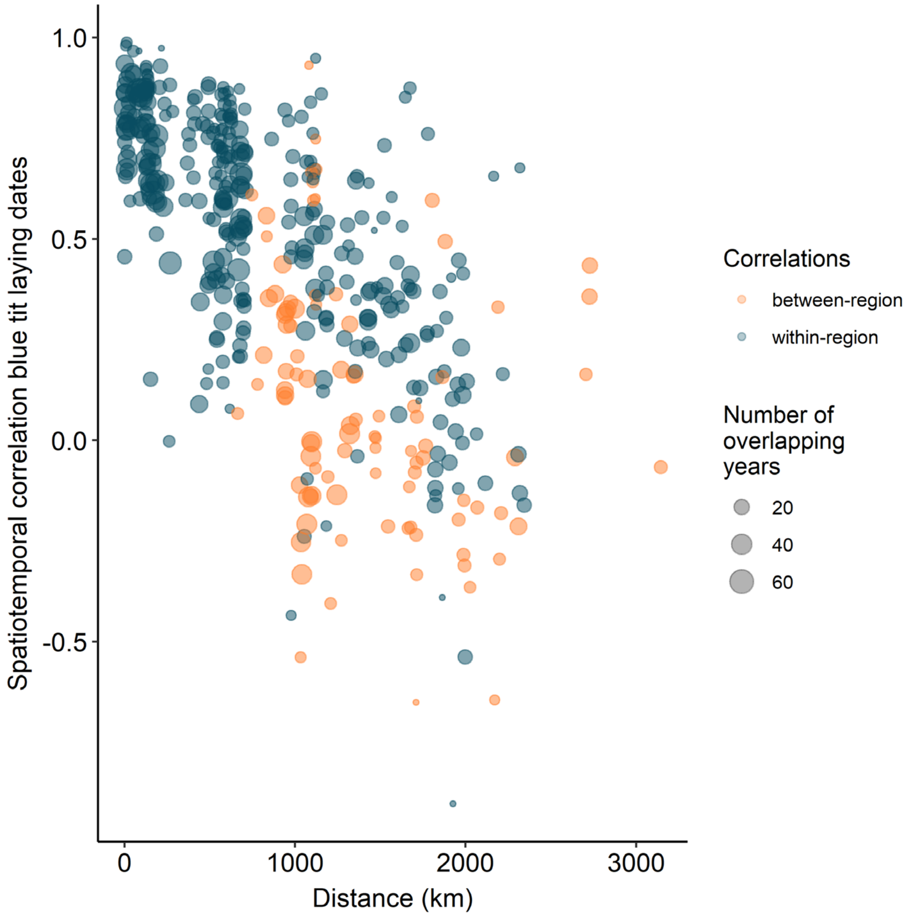

Figure S4. Pairwise correlations between the median laying date time series of blue tit populations in relation to the distance (in km) between them. Within-region pairwise correlations (i.e., northern populations in relation to other northern populations (≥ 45° N) and southern populations in relation to other southern populations (< 45° N)) are given in blue. Between-region pairwise correlations (i.e., northern populations in relation to southern populations and southern populations in relation to northern populations) are given in orange. Point size is proportional to the number of overlapping years between the pair of populations.
